# High impedance faults detection in power distribution networks using rogowski coils, kalman filtering, least-squares and non-recursive DFT computation engines

**DOI:** 10.1371/journal.pone.0320125

**Published:** 2025-04-17

**Authors:** Ziad M. Ali, Mostafa H. Mostafa, Shady H. E. Abdel Aleem, Ehab M. Esmail

**Affiliations:** 1 Electrical Engineering Department, College of Engineering at Wadi Addawaser, Prince Sattam bin Abdulaziz University, Wadi Addawaser, Saudi Arabia; 2 Electrical Department, Faculty of Engineering, Al Ryada University for Science and Technology, Sadat City, Egypt; 3 Department of Electrical Engineering, Institute of Aviation Engineering and Technology, Giza, Egypt; 4 Mechatronics Engineering Department, High Institute of Engineering and Technology, Elmahala Elkobra, Egypt; SRM Institute of Science and Technology (Deemed to be University), INDIA

## Abstract

This paper presents a novel computational engine based on non-recursive discrete Fourier transform (DFT) technology to detect high-resistance faults (HRFs) in power distribution networks. The non-recursive DFT approach utilizes the interconnection between a sliding window for current signals and a foundation function window during transient error. This non-recursive DFT technology is characterized by a fixed error in amplitude calculation and a rotated output angle. The proposed technique is compared against several established methods for high-resistance fault detection in distribution systems, including current reconstruction (CR) using Rogowski coils, Kalman filtering, and least-squares computational engines. The performance of each technique is evaluated by assessing the estimated percentage error in the calculation of fundamental and harmonic amplitudes. To study the proposed technique, the aforementioned methods were carefully modeled and simulated using MATLAB software for the IEEE 33-bus test feeder, simulated arcing faults, and Rogowski coils under various test conditions. The comparison is conducted under the influence of different arc models in the distribution system to assess the performance of the proposed technology. The comparative results demonstrate the effectiveness of the proposed non-recursive DFT-based technique in detecting high-resistance faults in power distribution networks, outperforming the other established methods considered in this study.

## 1. Introduction

### 1.1. Background and motivation

High-impedance faults (HIFs) pose a significant challenge in power distribution networks. HIFs occur when an electrical conductor contacts a highly resistant material during a fault, leading to a secondary fault current. These faults can be categorized into two types: active and passive. The passive type of HIFs results from the gradual deterioration of underground cable insulation over time [1]. The active type of HIF appears when an overhead conductor breaks and touches a high-resistance ground, generating an immediate temporary arc [2]. Values of currents associated with these faults are slightly lower than the normal load current, rendering conventional overcurrent relays ineffective for their detection [3]. Moreover, earth-sensitive relays are inaccurate during unbalanced load conditions [4]. The HIF current values can be influenced significantly by the type of conductive medium and its humidity level.

In such cases, the fault current is too small relative to the normal load current, resulting in conventional overcurrent relays failing to detect these faults. Traditional relays have been reported to be blind to approximately 80% of HIFs in the distribution system, leading to uncertainty in the effectiveness of protection schemes against HIFs [5]. Due to this undetected situation, HIFs pose a significant risk to public safety, as a dropped conductor can inflict dangerous shocks or cause fires through accidental human contact [6]. Furthermore, the damage inflicted on facilities by HIFs is viewed as a threat to the facility’s assets and could result in irreparable damage [7]. HIFs exhibit different characteristics compared to the typical short-circuit faults, and this complexity is attributed to the following factors:

1. The low current magnitude can barely be distinguished from a regular change in a balanced load [8].
2. Discontinuous arcing paired with harmonics, as well as noise in measurable signals [9].
3. The asymmetry and randomness inherent in the fault path lead to fluctuations in the magnitude of the HIF current over successive cycles [10].
4. Nonlinearity in the voltage and current relationship between signals [11].
5. There can be a gradual accumulation, where the HIF current magnitude steadily increases over successive cycles until a steady state [12].

These unique characteristics of HIFs make them particularly challenging to detect and diagnose using conventional protection schemes, necessitating the development of advanced techniques for reliable detection and mitigation. Besides, accurate knowledge of faults is crucial for engineers to develop effective solutions.

### 1.2. Literature review

Several researchers have explored technologies that can detect, classify, and locate HIFs during their unobserved state. This subsection aims to review the latest methodologies used to diagnose HIFs.

#### 1.2.1. Conventional techniques.

One conventional technique involves using a primary balanced current transformer to measure the neutral current, which is then used as an input to a sensitive ground fault relay. This method is widely used in industry [13]. However, the presence of unbalanced loads and the inherent residual current in the system requires adjusting the relay’s tolerance rate to avoid annoying false trips, making it more challenging to detect HIFs. Differential protection schemes are known to be sensitive to HIFs, but their implementation in distribution networks is complex due to the presence of multiple generating points and loading buses.

An alternative approach proposed in [14] involves installing a ground grid under the transmission line phases to capture a fallen conductor before it contacts a high-impedance surface. While this technique could potentially detect HIFs, it is not economically feasible due to the additional infrastructure required for the ground grid.

Applied industrial techniques like broken conductor detection, ground wire grid technique, and watt-metric protective relaying have also been introduced [14]. These methods rely on the overcurrent relay’s ability to detect the fault once the conductor is connected to the network. However, this approach may not be practical for all scenarios because it requires an additional ground grid installed on the poles of the long transmission line. The approach proposed in [15] involves putting a mechanical hook under the phase conductors connected to the neutral. If the conductor is downed, this method aims to trigger a short circuit from the line to the neutral line, leading to the overcurrent relays isolating the line. The conventional techniques discussed have limitations in accurately detecting and diagnosing HIFs. This highlights the need for innovative solutions and advanced methodologies to address the challenges posed by HIFs.

#### 1.2.2. Signal processing methods.

Signal processing techniques have been explored to analyze electrical currents during HIFs with nonlinear loads. A fast method based on the Fourier transform was introduced to investigate electric currents in 1φ feeders [16]. This method examines the even and odd harmonic components to evaluate the condition of the electrical distribution system. Analyzing the magnitude of the harmonics over time shows that the spacing between the 3^rd^ to 7^th^-order harmonics is distinct during HIFs, allowing its detection. However, this approach is sensitive to noise and requires noise reduction techniques to achieve the intended results. Another proposed method utilizes the Stockwell transform (ST) and measures the continuous phase angle of the 3^rd^ harmonic of the current signal [17]. The difference in the 3^rd^ harmonic is linked to line loading and switching, and a consistent 3^rd^ harmonic value suggests the presence of HIF. However, this method may take up to 150 ms to detect the fault, which could allow the power to increase the incident before it is identified. Hybridizing the maximum interference between discrete wavelets packet transform and empirical mode decomposition (EMD) was introduced in [18]. The probability of an existing HIF is indicated by this approach, which estimates the change in energy content between the inter-harmonic energy content in the fault signal and the pre-fault condition. However, not all HIF types may perform well under real-world operational settings, according to the model. A method has been proposed that uses the spectral density of the power calculated from the wave covariance matrix [19]. Discrete wavelet transformation is used to separate time information from frequency information, and the method uses wavelet transform (WT) to measure the third level of current signals. The calculation of the power spectral density of faulty current signals follows the analysis. This method uses threshold analysis as a framework for fault detection. However, it has yet to be validated to determine the precise location of the fault. Another technique to process 3φ voltage and current signals is based on the decomposition of the orthogonal component [20]. Under normal operating conditions, these calculated components maintain an absolute zero value, but they may experience variations during faulty conditions, resulting in HIF detection. Although this method’s fault detection capability is strong, in half of the studied scenarios, the absolute error in fault distance estimation is greater than 10%.

#### 1.2.3. Mathematical-based methods.

In the literature, researchers have made various advancements in the detection and analysis of HIFs in power distribution networks using mathematical-based methods. The key contributions include the extension of HIF detection methods to include mathematical and mechanical techniques [21]. This includes the introduction of a new low-frequency relay based on the pattern of the 3^rd^ and 5^th^ harmonic components of the high-resistance arc fault based on determining the time domain (pattern shape) of the harmonic voltage, current, power, and voltage/current ratios at these harmonic orders [22]. Creation of a differential equation-based technique to calculate the zero-sequence capacitance both upstream and downstream of the fault, allowing for the quick, noise-free, and self-calibrated detection of HIFs in isolated neutral networks [23,24]. Actual data can be used to estimate the fault admittance of medium-voltage systems, enabling the identification and localization of HIFs with resistances between 100 and 200 kΩ. A modified state estimation model for HIF diagnosis, incorporating voltage and power measurements, was introduced, but with significant errors during load variation [25]. An iterative approach to fault location requires a lot of computer processing power and uses the weighted least squares method to assess fault reactance and calculate resistance [26]. Using linear regression of the calculated fault distance components, an analytical estimator of the weighted least squares state is used to compute the fault voltage and current to identify HIFs in distribution networks [27]. A method estimating fault location by comparing fault parameters calculated from voltage and current measurements with reference data, but with a high computational burden and limited to single-feed distribution lines [28]. The representation of a time series of signal samples for HIF detection using linear prediction without taking nonlinear loading impacts into account [29]. These advancements demonstrate the ongoing efforts to improve the detection, analysis, and localization of HIFs in power distribution networks, enhancing grid reliability and safety.

#### 1.2.4. Artificial intelligence (AI)-based methods.

Three main steps are involved in AI-based methods for diagnosing HIFs: gathering data, extracting features through signal processing methods, and training with machine learning (ML) algorithms. The most recent developments in each of these procedures will be covered in this section.

***Data acquisition:*** The intelligent methods for diagnosing HIFs rely on the type of measurement signal used [30]. These current measurements have been employed to detect and classify HIFs in distribution networks, even though current waveforms in HIFs have harmonic components that are noticeable from normal load states [31]. Pay attention to how the percentage errors of current transformers impact current measurements. In HIFs, arcing phenomena cause an occasional voltage spike, particularly when contact with objects like trees causes the malfunction. Variations in fault resistance will arise from the motion-induced introduction of air gaps between the conductor and the surface. A technique based on arcing voltage measurements has been discussed in [32]. However, voltage changes make capturing the waveform increasingly challenging because the voltage is dipped. Consequently, to improve findings, neural network (NN) training was used for both current and voltage waveforms in order to diagnose HIFs in distribution systems [33]. Unfortunately, this method expands the dataset for the neural network, necessitating a thorough study to restrict training on important features and manage the computational burden. It has been determined that the HIF problem is a pattern classification challenge that NN classifiers might face when training using features taken from measurements of magnetic field strength, voltage, and current [34]. The method of using resistance measurements to diagnose HIFs has also been introduced, where the resistance values are compared with the prime impedance values of the transmission line under normal operating conditions [35]. This comparison can shorten network outages and shed light on the line’s fault location. Resistance, however, is unable to adequately capture the nonlinearity, asymmetry, or arcing characteristics of HIFs, and it is severely compromised when attempting to identify such faults with these measurements. The synchronous phase-measurement units aim to employ a signal in a set period as an absolute value paired to the phase angle [36]. These measurements accurately represent voltage and current waveforms in power systems. The use of synchronous phase measurement units in HIF diagnosis has been discussed [37]. However, more research is needed to determine how to use these units for HIF diagnosis while taking fault asymmetry and nonlinear load into account. At present, low-power instrument transformers and sensors have become prevalent in power systems. This development is a result of the improved network management and control operations made possible by these new generations of instrument transformers [38]. Although not a novel concept, the Rogowski coil (RC), invented in the 19th century by Walter Rogowski, is one of the most widely used low-power instrument transformers. Its numerous applications are made possible by the developments in digital systems, signal processing, electronics, and other fields. Owing to its numerous benefits—lightweight, inexpensive, flexible, linear, immune to core saturation (because its core is composed of a non-magnetic material), and an easy-to-use design that allows it to be applied in a variety of RC settings has become increasingly popular. On the other hand, positional errors indicate that some RC measurements deviate from expected values. Nevertheless, achieving optimal coil design can reduce these errors to less than 0.1% [39]. Therefore, the design of the RC can be further improved using new optimization techniques, such as particle swarm optimization or others, to achieve the desired performance [40]. Another key consideration of the RC is its susceptibility to external magnetic fields generated by extraneous currents. However, this issue can be effectively mitigated through the use of shielding or by employing a design with two opposing coils, which helps minimize the impact of these external magnetic fields [41]. Furthermore, the RC differs considerably from the conventional current transformer. Traditional current transformers directly convert the input current to a lower current within their secondary coil, with the conversion ratio determined by the transformer design. However, at its output terminals, the RC generates a low-voltage signal. The reconstruction current, which is the intended sensing current, must then be obtained by specific processing of this voltage signal. Interestingly, research has also investigated the influence of geometric parameters on the high-frequency performance of the RC, particularly in the context of partial discharge measurements [42]. Furthermore, research has examined the behavior of the RC in relation to the position, form, and course of the power conductor as well as the influence of a non-circular coil geometry and the presence of external currents [43]. Finally, it is worth noting that low-cost RCs and their associated auxiliary circuits have found application in the measurement of power frequency quantities [44].

***Feature extraction:*** Signal processing techniques must be used to extract features so that machine learning (ML) algorithms can operate effectively. The Fourier transform is one such method that is frequently used in applications addressing power quality disturbances. This method finds signal frequency components when there are disruptions [45]. Although the Fourier transform is continuous over time, it is frequently used in discrete forms in computational applications, such as in the detection of HIFs [46]. Fast Fourier transform (FFT), another form, was also employed [47]. Only the features in the frequency range can be represented by the Fourier transform for the purpose of applying the HIF diagnostic. WT is a sophisticated method for signal processing that can represent information in both the time and frequency domains, in contrast to Fourier transform [48]. Furthermore, compared to discrete wavelet transforms, wavelet packet transform (WPT) conversions offer more information [49], as they can degrade in higher and lower frequency bands at each level. This application has given adequate results in the HIFs’ detection and classification of [50]. Furthermore, the application of multi-WT is discussed in [51]. Several scaling functions and associated multi-wavelets cause the threshold to be exceeded in the multi-WT, which is an extension of the scalar wavelet. As a result, the node will create a value product that is either equal to or close to one; if not, it will have zero.

***Machine learning:*** A neural network (NN) is a complex framework made up of many nodes that have undergone exact processing to carry out several demanding mathematical operations. The strength of the network administers these operations, referred to as weight, which is influenced by external inputs known as bias. This intricate process produces a set of historical patterns through the iterative mechanism of adaptation [52]. Typical artificial neuron inputs have signals weighted through multiplication factors and are aggregated along with the bias to feed a node. Following that, the value is compared to a predefined threshold. If the result matches or exceeds the minimum, the node will return a value that is almost identical to one; if not, it will provide a value of zero. NN training is mostly concerned with identifying the best weights and biases to achieve the desired results. The multi-perceptron-based model was employed to diagnose HIFs through the effective utilization of backpropagation technology. This approach trained the network to accurately detect and classify faults [53]. However, it has become evident that a more sophisticated technique is required to gather the suitable number of hidden layers and neurons to develop the most prominent results with reduced computational time; hence, a hybrid approach using the multi-perceptron in conjunction with the regression of the Gaussian process was implemented [54]. The multi-perceptron was utilized to find the optimal results (weights and biases) for the detection and classification of HIFs. At the same time, the Gaussian process regression was a linear regressor aimed at approximating the location of the fault within the transmission line. The ST has also been used as a preliminary processing and feature extraction technique to develop the most efficient approach [55].

#### 1.2.5. Rogowski coil for sensing faults.

Researchers have suggested the use of RC in many applications, such as measuring power frequency current [56], pulse and pulse current measurement [57], detecting the fault in converters [58], tracking the condition of the high-voltage insulators [59], power network equipment [60], smart meters [61], and other applications. Power frequency current and various impulse current properties of single-core and multi-core cables are measured with it. Power frequency measurement has various applications, one of which is fault diagnosis. In [62], an RC model specified using the system identification toolbox is used to obtain the exact terminal voltage, and the transient slope of the ground and aerial voltage during the fault is extracted to classify the fault type, distinguish the fault section, and identify the faulted phase in each cycle. In [63], it has been shown that RC can detect high frequencies and identify high-frequency transient faults in a variety of fault scenarios, such as a high resistance fault and intermittent fault cases, where RC measures and extracts the fault’s surges. The distance the fault surge traveled to reach the measuring point determined the sensitivity of each RC and the surge arrival time. In covered conductor lines, RC has also been effectively employed to measure and extract high-frequency partial discharge signals [64]. Distributed RC is a fault pass indicator used to identify the active travel wave fault location, where active travel waves are injected by switching the neutral point of unearthed and compensated networks using thyristors [65]. This method can create traveling waves even when the faulty feeder is in service. The results confirm the applicability of using RCs for traveling wave applications in fault locating and fault management in distribution networks.

### 1.3. Contributions and novelty

This work examines the computational engine utilized for detecting HIFs, which relies on a non-recursive DFT to measure the fundamental and higher-order harmonics within distribution networks. One of the modeling techniques selected for HIF was the numerical technique, which demonstrated accuracy in its modeling using MATLAB simulation software. Three computational engines were chosen to conduct an accurate comparison with the proposed engine, and this comparison revealed the proposed engine’s effectiveness in detecting HIF. The MATLAB simulation software modeled the IEEE-33 bus distribution feeder to facilitate this comparison. Furthermore, the comparison has been expanded to include recently published methods for HIF detection, as well as the computational engines under study. The simulation results demonstrated the proposed computational engine’s accuracy, reliability, and security in detecting this challenging type of fault. The novelty and key features of the proposed computational engine, which verify the effective achievement of its purpose in comparison to recently published studies, are (i) Function: The engine can accurately estimate all fundamental and higher-order harmonics; (ii) Cost: No new components are required, as the computational engine can be integrated into the pre-existing protective relay at the beginning of the feeder, in contrast to other existing schemes; (iii) Flexibility and autonomy: The engine does not rely on powerful threshold values and instead depends on the nature of its non-recursive DFT-based calculations; and (iv) Accuracy, reliability, and security: The engine offers advantages in terms of these standards compared to other existing schemes, as it provides distinct results for these criteria.

### 1.4. Organization

**Fig 1:**
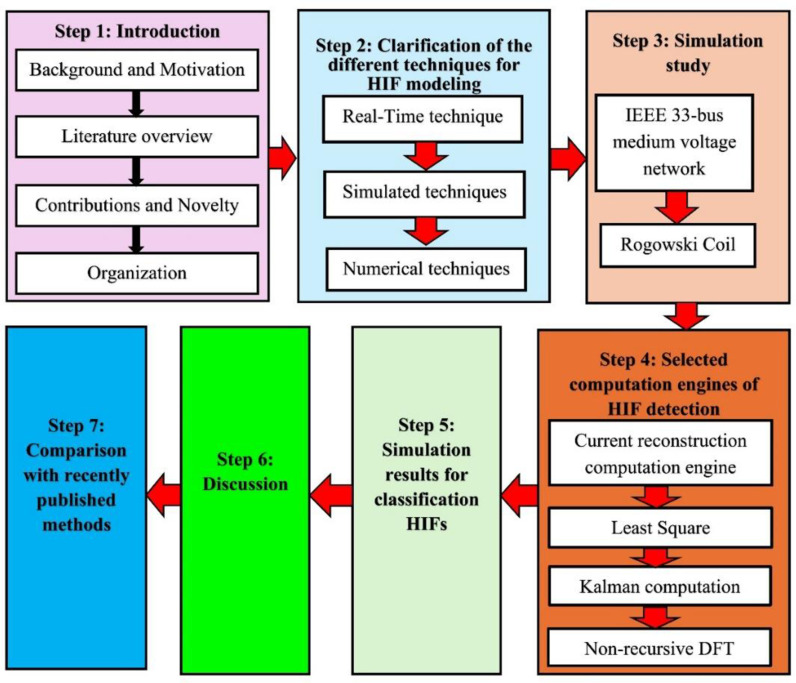
A schematic overview of the manuscript and the steps implemented for evaluating the proposed computational engine.

The remainder of the manuscript is organized as follows: Section 2 presents the HIF modeling methods found in the literature, and the most accurate modeling methods have been selected for inclusion. Section 3 details the modeling of both the IEEE 33 bus distribution feeder and the RC circuit used in one of the engines to detect HIF. In Section 4, four computational engines that can detect HIF are introduced. The results of the four computational engines are presented in Section 5. A comparison of the four computational engines is made in Section 6 to determine the proposed engine. Section 7 expands the comparison to include other methods from the literature to verify the efficacy of the proposed engine. Finally, Section 8 is devoted to the conclusions derived and future directions of the work. A schematic overview of the manuscript and the steps implemented for evaluating the proposed computational engine are depicted in Fig 1.

The innovation of the proposed engine and the important characteristics that prove its success in performing its function sufficiently compared to the previous methods are:

***Proposed method***

- Non-Recursive DFT Technology: A non-recursive DFT engine is used to estimate detecting fundamental and harmonic amplitudes, which processes current signals through a sliding window and a foundation function window. This engine aims to provide a constant error in the calculation of amplitude and a rounded output angle, which enhances detection accuracy.
- Integration with Existing Systems: This method can be integrated into existing protection relays without the need for additional components, making it cost-effective.
- Comprehensive Fault Detection: It accurately values both fundamental and higher-order harmonic harmonics, allowing for better identification of HIFs.
- Simulation and Evaluation: Modeling and simulating this engine using MATLAB, specifically for the IEEE 33 bus test feeder, under various conditions, including arc faults.

***Superiority over existing methods***

- Performance: The non-recurring DFT-based method has surpassed established techniques such as Kalman filtering and least-squares-square computational engines and is considered a competitive engine for current reconstruction using Rogowski coils in terms of accuracy and reliability in detecting HIFs.
- Error Reduction: Constant error in amplitude estimating reduces uncertainty, an essential problem in traditional methods that often struggle with low current amounts typical of HIFs.
- Flexibility and Autonomy: Unlike traditional methods that rely on predetermined threshold values, the proposed method adapts based on the nature of the error and its robustness against various fault conditions.
- Cost-Effectiveness: By incorporating existing systems and not requiring new hardware, the method provides a compelling alternative to other expensive solutions that require additional infrastructure.
- Adaptability to Various Conditions: Test engines against different arc models under different test conditions are performed, demonstrating their effectiveness across a range of scenarios.

The proposed non-recurring DFT-based engine represents significant advances in HIF detection, addressing existing method limitations by providing improved accuracy, cost-effectiveness, and adaptability. This positions it as a valuable tool to enhance safety and reliability in power distribution networks.

## 2. Clarification of different techniques for HIF modeling

Arc modeling includes the representation in different applications. They are either normal operations or abnormal conditions. These applications are as follows:

- Arc models representing dynamic loads such as arc furnaces [66].
- Current interruption, such as AC and DC circuit breakers for different voltage levels.
- Transient faults such as the cases presented in this section.

All these arc models are dynamics. According to the application type and conditions, the arc model parameters, including the arc elongation parameters, have been changed. This parameter has a significant effect on the arc voltage.

Many research works include modeling HIFs as a crucial component since the modeling approaches’ ability to reproduce HIF features accurately is what determines how accurate the results are. To effectively mimic these HIF qualities in a simulated environment—such as nonlinearity, asymmetry, unpredictability, intermittency, build-up, and shoulder characteristics—complex modeling techniques are needed. As a result, the modeling strategies currently employed in the literature to depict these difficult HIF characteristics will be addressed throughout this section.

### 2.1. Real-time technique

The ultimate objective of HIF diagnosis is solving an actual issue. Thus, a simple method that can be used is real data generated in a high-current research facility. In [22], a range of HIFs were simulated, and the corresponding current and voltage magnitudes were recorded using digital data recording equipment. Materials like tree branches, grass, and concrete surfaces were provided in both dry and wet circumstances. Owing to space restrictions, this method may only be feasible for some other researchers, even if it offers useful, practical data for research and is the closest approximation to genuine HIF. To replicate real HIF performance, laboratories will also require costly high-voltage equipment in addition to strict safety precautions to reduce the possibility of high-frequency arcing.

### 2.2. Simulated-based techniques

The fault conditions are simulated in a virtual environment as part of the second category of HIF modelling methodologies. This paper will describe several models, including those used in MATLAB, ATPDraw, and electromagnetic transient tools, that are used in the literature to mimic the characteristics of HIFs.

#### 2.2.1. Single variable resistance.

Using (1) to determine the arcing resistance (*R*_*arc*_), this model was first developed by simulating the arcing properties of HIF based on the concepts of Cassie [67] and Mayr [68].


Rarc=R0(1−e−tτ) (1)

where *R*_0_ is the initial fault resistance, and *t* denotes the timeand τ is a constant of the system. This method adds a degree of randomization to HIF simulations, but it misrepresents the fault’s asymmetry and nonlinear features.

#### 2.2.2. Variable resistance and single inductance.

**Fig 2:**
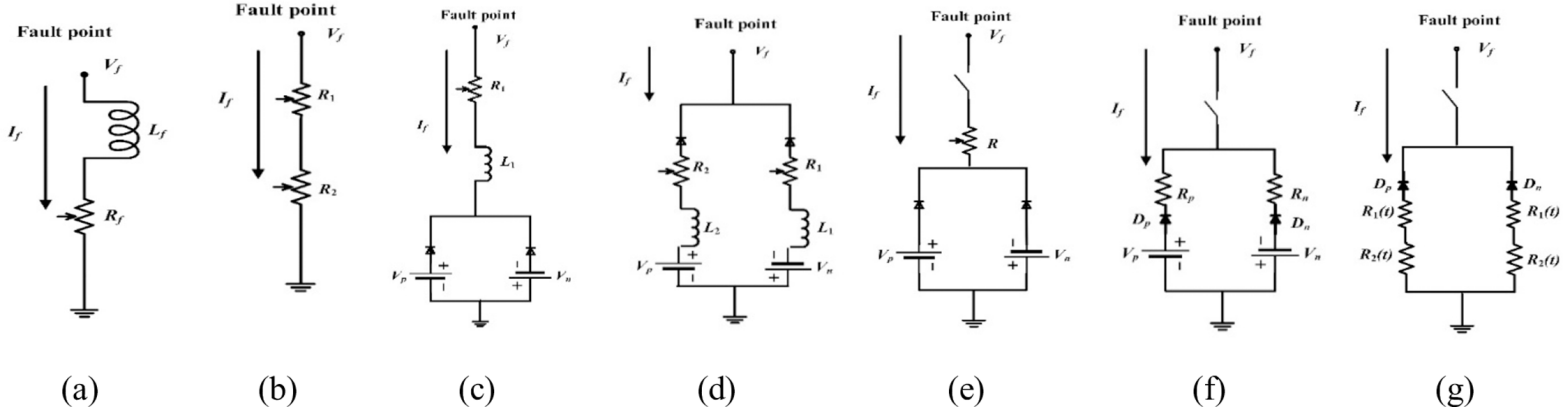
HIF modeling: (a) variable resistance with inductor model, (b) two variable resistance model, (c) two antiparallel diodes model, (d) two antiparallel diodes with resistances and inductors, (e) nonlinear resistance and two antiparallel diodes, (f) two nonlinear resistances and two antiparallel diodes, and (g) two time-varying resistances.


In [69], the HIF illustration is depicted in Fig 2(a). The fault resistance *R*_*f*_ is calculated using the following mathematical equation:

$$R_{f}=R_{0}\left(1+\alpha {\left(\frac{I_{f}}{I_{0}}\right)}^{\beta }\right)$$ (2)

where *α* and *β* are user-defined constants, *I*_*f*_ denotes the fault current, and *I*_0_ denotes the initial fault current value. An inductor with a typical value of *L*_*f*_ =  3 mH is connected in series with the fault resistance [70]. The approach is simple to use and has a low computational burden overall. However, it is far from an accurate representation of actual HIF data due to the empirical assumptions made in the model.

#### 2.2.3. Two variable resistances.

A good method for modeling the shoulder characteristics, build-up, asymmetry, and nonlinearity of HIFs was introduced in [71] through the use of two variable resistors, as shown in Fig 2(b). *R*_1_ is designed to model the asymmetry and nonlinear characteristics of the HIF current *I*_*f*_ and voltage *V*_*f*_, with values sampled over time. The build-up features are overlooked because the obtained values of *R*_1_ are from cycles with amplitudes that are comparable to those of prior cycles. Conversely, *R*_2_ is intended to reflect the HIF’s build-up and shoulder features. The fault current will eventually attain a steady state value since the resistance will start high and gradually drop. The sampled data may differ based on the type and state of the surface. Furthermore, this model does not take the discharged arcing component into account [72].

#### 2.2.4. Two antiparallel diodes.

Another model was introduced in [73] to represent the distinctive characteristics of HIFs, as depicted in Fig 2(c). In this model, the resistances *R*_1_ and *R*_2_, together with inductances *L*_1_ and *L*_2_, add the nonlinear property to the HIF. Additionally, the factors of *V*_*p*_ and *V*_*n*_ in the discharged arc voltage of the incident are incorporated. This model is designed with directional diodes so that if *V*_*f*_ >  *V*_*p*_, the fault current will flow from the source to the ground. The opposite will occur at *V*_*f*_ <  *V*_*n*_, where current will flow back to the source, and when *V*_*n*_ <  *V*_*f*_, no current will flow into the system. Building upon this model [74], expanded on Emmanuel’s model through a variable resistance experiment, as depicted in Fig 2(d). However, this model cannot simulate the build-up and shoulder features of HIFs.

#### 2.2.5. Nonlinear resistance and two antiparallel diodes.

In [75], a new HIF model was introduced, consisting of a nonlinear resistor, two diodes, and two DC sources that randomly change the amplitude every half cycle, as shown in Fig 2(e). This model is designed to represent the specific dynamics and randomness of HIFs. The mean change and standard deviation of the DC source voltage amplitude are used to closely approximate the characteristics of different ground surfaces, such as asphalt, sand, and grass.

#### 2.2.6. Two nonlinear resistances and two antiparallel diodes.

The high-resistance fault model introduced in [5] and shown in Fig 2(f) is identical to the source diode resistance model. It consists of two DC sources, *V*_*p*_ and *V*_*n*_, connected by two diodes, *D*_*p*_ and *D*_*n*_, respectively. The DC sources have asymmetric magnitudes, and their random values vary around *V*_*p*_ and *V*_*n*_ every 0.1 ms. This model captures the unequal nature of the fault current and the disappearance of the middle of the arc. The values of *V*_*p*_ and *V*_*n*_ rely on the system voltage to perform the simulation and the amount of asymmetry that can be modeled. If *V*_*ph*_ >  *V*_*p*_, the corresponding current flows towards the earth and is reflected when *V*_*ph*_ <  *V*_*n*_. When *V*_*n*_ <  *V*_*ph*_ <  *V*_*p*_, no current flows. The variance of *V*_*p*_ and *V*_*n*_ values adds randomness to the number of asymmetries and the arc’s disappearance period. Variable resistances *R*_*p*_ and *R*_*n*_ are also included in series with the diodes, alternating separately and randomly every 0.1 ms. The model parameters used for simulation are *V*_*p*_ =  1.0 kV with a random difference of ± 10%; *V*_*n*_ =  1.0 kV with a random difference of ± 10%; *R*_*p*_ =  random difference with 100–150 Ω; and *R*_*n*_ =  random difference with 100–150 Ω. This model is designed to restrict the fault current to always be less than 10% of the total load current of the feeder.

#### 2.2.7. Two time-varying resistances.

The improved method, introduced in Fig 2(g), consists of two time-variable resistors, *R*_*n*_(*t*) and *R*_*p*_(*t*), that are controlled by the transient analysis of an electromagnetic transient program control system. This method is designed to model the HIF arcing characteristics more accurately. The key aspects are:

1. One time-variable resistance, *R*_*n*_(*t*), uses the nonlinearity and asymmetry of the voltage-current (*V*-*I*) property to represent these aspects of the HIF.
2. The other time-variable resistance, *R*_*p*_(*t*), is used to formulate the build-up and shoulder characteristics of the HIF [34].

To better capture the complex and dynamic behavior of HIFs, this amended method makes use of these two time-variable resistors controlled by transient analysis.

### 2.3. Numerical techniques

**Fig 3:**
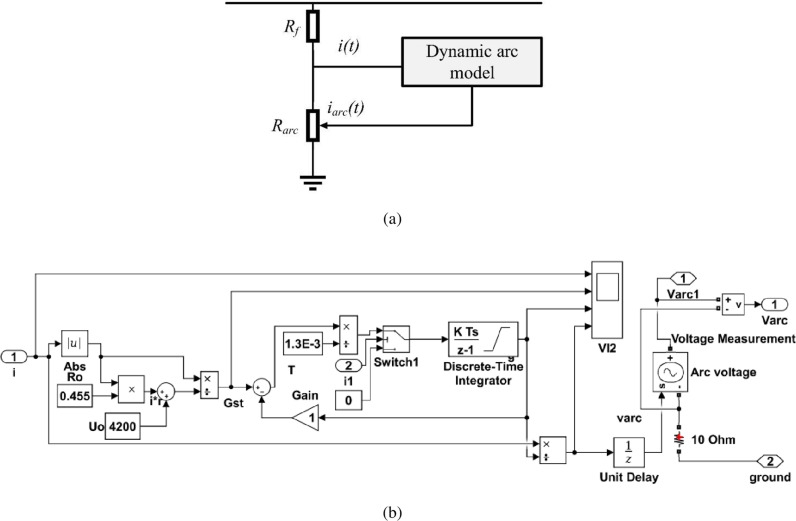
Arc model: (a) structure, and (b) the Simulink tools used to simulate the arc model.

Many numerical models have been developed to describe the behavior of arcs. Most of these models have been utilized for circuit breaker arcs [76], and several have been applied to long arcs [77] or arcing faults [78]. The most extensively used models are those based on the assumption of thermal equilibrium. The thermal model boasts the longest history among dynamic arc models, tracing its origins back to the work of Cassie in 1939 and Mayr in 1943 [79]. They provided the initial description of arc conductivity as a first-order differential equation. Over time, these dynamic equations have been optimized and modified to enhance model validity and reduce computational requirements. Notably, the arcing fault [78] is represented by a successive differential equation, as demonstrated in the following expression.


$$\frac{dg}{dt}\;=\frac{1}{\tau }\;\left(G-g\right)$$ (3)

where *t* is the time, *τ* is the arc time constant, *g* denotes the instantaneous arc conductance, and *G* is the stationary arc conductance, which is defined as follows:


$$G\;=\;\frac{\left|i_{arc}\right|}{\;V_{arc}}$$ (4)

where *i*_*arc*_ is the instantaneous arc current, and *V*_*arc*_ is the stationary arc voltage. The arc time constant is defined as:


$$\tau \;=\;A\;\cdot \;e^{Bg}$$ (5)

where *A* and *B* are constant parameters representing the experimental values of the positive and negative half cycles. However, the appropriate parameters for the half cycle do not provide good agreement in properties during the negative half cycle. The parameters were set to match the results of the experimental arc current introduced in [80]. The dynamic arc model per instantaneous arc current *i*_*arc*_ is solved by determining the arc parameters related to the Kizilcay arc modeling approach described in [78]. As seen in Fig 3(a), the fault resistance *R*_*f*_ found in the HIF model is a linear resistance representing the fault path resistance through an object with high impedance (was a tree in that study). Fig 3(b) depicts the Simulink tools in the MATLAB platform used to simulate the arc model, where the resistance representing the arc is variable type, and its value is obtained through the inverse of the instantaneous arc conductance, which aligns with the Kizilcay arc model. Also, the dynamic arc model per *i*_*arc*_ expressed in (3) was adapted to represent the long arcs.

**Fig 4:**
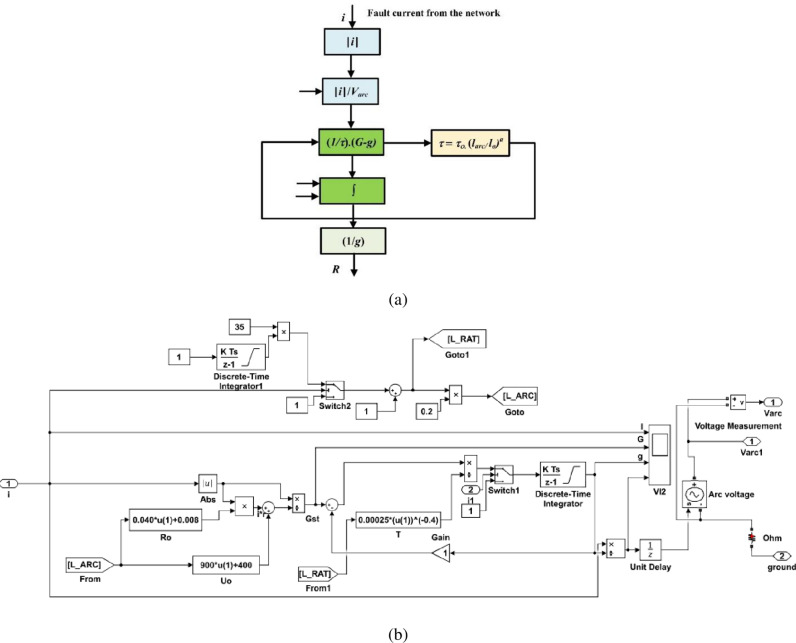
Arc model: (a) long arc representation, and (b) the Simulink tools used to simulate the long arc model.


As seen in Fig 4(a), the arc parameters are determined such that the arc time constant is 1.3 ms, the arc voltage per unit length is 12 V/cm, the arc resistance per unit length is 1.3 Ω/cm, and the arc length is 350 cm, as reported in [81]. Similarly, Fig 4(b) depicts its block diagram used to simulate the long arc model. The implementation of this arc model is suitable only for the primary arc period with characteristics of high arcing current. However, for the secondary arc period, the arcing current is significantly reduced due to the opening of the single-pole breaker at both terminals of the faulted phase, as discussed in [82], in terms of *U*_0_ denotes the characteristic arc voltage, and *r*_0_ denotes the characteristic arc resistance.

$$G=\frac{\left|i_{arc}\right|}{U_{0}+r_{0}.i_{arc}}$$ (6)

The parameters *U*_0_, *r*_0_, and *τ* depend on the arc length (*l*_*arc*_) and are calculated using the following equations derived from arc measurements.


$$U_{0}=0.9\left(kV/m\right)\;l_{arc}+0.4\left(kV\right)$$ (7)

$$r_{0}=40\left(m\Omega /m\right)\;l_{arc}+8\left(m\Omega \right)$$ (8)

τ = τ0 ⋅ larcl0α (9)

where *τ*_0_ is the initial time constant, *l*_0_ is the initial arc length, and *α* is the negative value coefficient. In this work, the numerical model is utilized to distinguish the high-accuracy performance characteristics obtained from it.

## 3. System under study and Rogowski coil model

### 3.1. IEEE 33-bus medium voltage network

**Fig 5:**
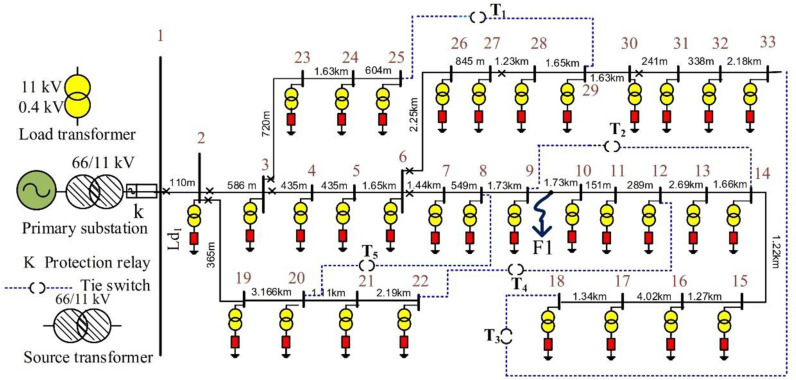
IEEE 33-bus test feeder.

The IEEE 33-bus 11 kV radial distribution feeder was selected as the study system. This feeder data was obtained from [83] and is presented in the S1 Table. Fig 5 illustrates this feeder, depicting a single-line diagram of the modified feeder with added source and loading adaptors. A single circuit breaker is inserted at the beginning of the feeder, and 64 normally closed switches are positioned at each end of a section, as depicted in Fig 5. In addition, the system has five normally open switches (also known as tie switches) to allow for system reconfiguration as described in [84]. The positive sequence data for the feeder parameter and loads characterize the test feeder.

MATLAB software was utilized to simulate the selected distribution feeder. Each feeder section was modeled using the π circuit representation in the Simulink tool. Each load was represented as a 3φ balanced load implemented through a set of RLC branches. Appropriate remote-control switches and communication systems were added to enable the feeder automation and fault management process. Finally, measurement units were already in the protection relay at the primary substation and each feeder lateral exit. A sampling rate of 200 samples per cycle was selected. The discrete recursive Fourier transform (DRFT) was employed to estimate the values associated with the fundamental currents and each harmonic, serving as a baseline reference for the methods presented in the following sections.

### 3.2. Rogowski coil model

**Fig 6:**
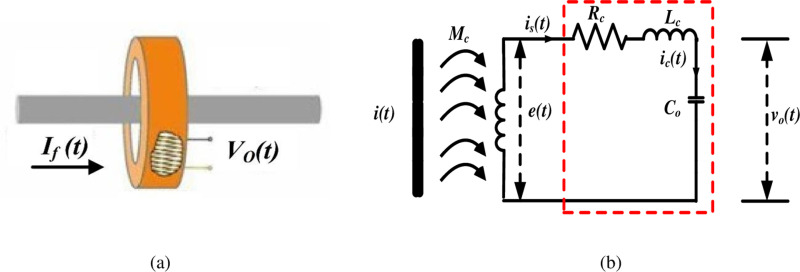
RC model: **(a)** RC is placed around a current-carrying conductor, and (b) lumped-parameters equivalent circuit of the RC.


Initially, the RC is placed around a current-carrying conductor, as shown in Fig 6(a). The current that passes through the conductor is distorted with both low- and high-order harmonic components. The fault current can be expressed as follows:

$$i_{f}(t)\;=\;I_{m}\sin\left(\omega t\pm \theta \right)\;+\;\sum_{2}^{n}{Ι}_{mn}\sin\left(n\omega t\pm \phi_{n}\right)$$ (10)

where *I*_*m*_ denotes the maximum fundamental current component (A), ω stands for the fundamental angular frequency component (rad/s), *t* represents the time in seconds, *θ* denotes the fundamental phase angle component (rad), *I*_*mn*_ stands for the maximum *n*th harmonic current component (A), *θ*_*n*_ is the *n*th harmonic phase angle component (rad), and *n* represents the harmonic order. This expression captures the fundamental current component as well as the summation of the *n*th harmonic current components, which are necessary to fully represent the fault current waveform. The induced electromagnetic forces are generated in the coil with fundamental and harmonic components. The induced electromagnetic forces of Faraday’s law can be calculated as follows:


$$e(t)=M_{c}\frac{di}{dt}$$ (11)

where *e*(*t*) denotes the EMF generated in the RC by induction (V), *M*_*c*_ denotes the mutual inductance (H), and *i* is the current passing through the RC (A). Substituting equation (10) into equation (11) provides the following expressions for *e*(*t*) and the output voltage at the RC terminals *v*_*o*_(*t*):


$$e(t)\;=\;M_{c}\omega \left[{Ι}_{m}\cos\left(\omega t\pm \theta \right)\;+\;\sum_{2}^{n}{Ι}_{mn}\cos\left(n\omega t\pm \phi_{n}\right)\right]$$ (12)

$$v_{o}(t)\;=V_{om}\sin\left(\omega t\pm \beta \right)\;+\;\sum_{2}^{n}V_{omn}\sin\left(n\omega t\pm \beta_{n}\right)\;$$ (13)

where *V*_*om*_ denotes the maximum fundamental output voltage component (V), *β* denotes the fundamental phase angle component of the output voltage (rad), *V*_*omn*_ denotes the maximum *n*th harmonic voltage component (V), and *β*_*n*_ is the *n*th harmonic phase angle component (rad).

In practice, especially at higher frequencies, the output voltage at the RC terminals varies in both magnitude and phase angle from the EMFs. This is brought about by the RC’s resistance (*R*_*c*_), coil capacitance (*C*_o_), and self-inductive effect (*L*_*c*_) [56]. As stated in [56], an external resistance was previously used at the RC terminals to properly dampen the high-frequency components in the induced EMF.

In this work, the coil stray capacitance *C*_o_ is used as a substitute for the terminal resistance, as shown in Fig 6(b). The rationale for this choice is to obtain an output voltage signal with significantly reduced very high-frequency (noise) components while minimizing the difference between the output and the induced voltages.

The self-induction, in combination with the coil stray capacitance *C*_o,_ acts as a second-order low-pass filter. This configuration enables more accurate digital processing, especially with a low sampling time. By using the coil stray capacitance in this manner, the output voltage waveform can be conditioned to better reflect the induced EMF, facilitating improved signal processing and analysis.

The performance of the RC can be verified through accurate modeling, which is an essential requirement. Such modeling approaches can be classified into three main categories: models based on frequency measurements [85], lumped models [86], and distributed models [87]. This work develops a simulation model to validate the measuring process using a lumped modeling approach. For this, the MATLAB Simulink tool is employed. Using equations (11) and (14): The RC is simulated in accordance with the lumped equivalent circuit depicted in Fig 6(b).


$$e(t)\;=R_{c}\left(i_{s}\right)+L_{c}\left(\frac{di_{s}}{dt}\right)+\frac{1}{C_{o}}\int i_{s}dt$$ (14)

**Fig 7:**
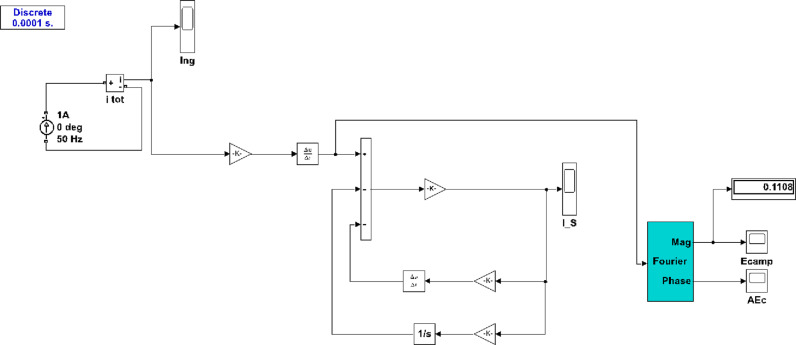
Modeling the RC in Simulink.

By employing the lumped modeling technique, the behavior of the RC can be accurately represented and analyzed. This simulation-based validation ensures that the mathematical formulation and the underlying assumptions are sound, providing confidence in the measurement process and the subsequent analysis. Hence, the simulation is carried out as shown in Fig 7.

Several RC simulators have been used to measure fundamental current values and harmonics, with parameters such as *R*_*c*_ =  2.4 Ω, *L*_*c*_ =  1.2 mH, *C*_*o*_ =  10.3 nF, and *M*_*c*_ =  1.8 μH [88]. These parameters were used to measure fundamental current and low/high harmonics. A practical RC coil was designed and manufactured in [56] with several 235 turns, *R*_*c*_ =  0.3 Ω, *L*_*c*_ =  1.345 mH, *C*_*o*_ =  67.569 pF, and *M*_*c*_ =  35.975 nH. Decreasing turns and smaller core diameters increase performance and resonant frequency, making it more suitable for higher frequency measurements. Other RC coils have been modeled in [89,63]. The rectangular shape of the RC for high-sensitivity purposes was suggested in [89], with *R*_*c*_ =  0.1922 Ω, *L*_*c*_ =  2.5 μH, *C*_*o*_ =  41.21 pF, *M*_*c*_ =  4.6825 nH, and the resonant frequency of this coil is 3.4 MHz. In [63], which has 1900 turns and an outer and inner diameter of 109 and 101 mm, respectively. Based on these values, the parameters of the equivalent circuit were *R*_*c*_ =  63 Ω, *L*_*c*_ =  0.191 mH, *C*_*o*_ =  51.6 pF, and *M*_*c*_ =  300 nH. This means that the RC parameters can be optimized for different performance requirements, particularly relating to frequency response and sensitivity.

## 4. Selected computation engines for HIF detection

### 4.1. Current reconstruction computation engine

Various computation engines are used for reconstruction. First, reconstruction engines to measure high pulse current have been developed. The standard computing engine was implemented to reconstruct the input current of RCs using an analog integration circuit at the output terminals [90]. At low frequencies, this integral-based approach was used because RC circuits perform as differentiators. The RC circuit and its integration circuit worked together to produce a rebuilt signal of low magnitude, which resulted in low sensitivity. On the other hand, increasing the output signals also increases the noise signals, which presents a significant problem for high-frequency applications. In [91], the authors consider the RC design in its form of integration. However, this method was limited to high-frequency applications. As mentioned in [64], a calibration-based method has been presented to reconstruct the current of an RC by monitoring the coil’s output voltage for a specific pulse and dividing the maximum value of the transmitting and receiving pulses. This method yields a constant calibration coefficient of the output voltage. This calibration-based method does not reconstruct the entire frequency spectrum’s output voltage; rather, it just reconstructs the measured signal’s peak value. Computation engines have also been introduced to reconstruct the current of the RC in either domain of time or frequency. The time-domain engine employs implicit model-based numerical integration on samples of time-domain data. Even this, the engine is dependent on the coil’s physical structure and has a restricted verification range relative to the coil’s complete range. The inverse transfer function is the basis of the frequency-domain engine and plays a major role in determining its performance. Nevertheless, this engine’s precision is restricted to the designated frequency range [92].

In order to minimize the phase difference between the measured current and the associated terminal voltage, the integration circuit has been improved in the coil design based on the frequency response [93]. This coil’s performance has been compared to that of traditional current transformers for protective relay applications. It can be utilized for both protective and measurement devices in low-frequency applications [94]. DFT computation engine was introduced to reconstruct the fundamental AC component of the power frequency [56]. In [95], a differential computation engine was introduced to reconstruct the current. It is only appropriate for measuring the fundamental power component. A computational engine was introduced to reconstruct AC voltage with power frequency [96]. The proposed computational motor uses a low-capacity capacitor and a double-wound RC. This computational engine is based on measuring the capacitor’s current using the RC double wound. The voltage is reconstructed via the capacitor connected in parallel with the system at which the AC voltage is intended to be measured. A non-invasive design of the RC for measuring three-phase currents through a three-core cable was utilized in [97]. This design is characterized by no need to remove the insulation layers of the 3-core cable to measure its core currents. The certified RC design uses three separate coils wound in the same non-magnetic circular core with equal spacing between them. The three induced EMFs are used to reconstruct the current passing through each cable core using a current reconstruction (CR) technique based on the basic concepts of the RCs working theory. Three-phase motor currents passing through a three-core cable were measured using a dual-coil RC [98]. A dual-coil RC is mounted externally around a three-core cable without removing its insulation layers, reducing the measurement’s complexity. Dual coils were mounted around a non-magnetic circular core, taking 120° between the center points into account. The EMFs induced in each coil due to the magnetic flux linkage between the coils and cable cores are used to reconstruct three-phase motor currents through the cable cores. The cable currents were reconstructed using the reconstruction computational engine introduced in [97] and developed in [98].

The authors of this work employed a computational engine to reconstruct, particularly at high frequencies, keeping in mind the previously stated caution that the output voltage should not be measured instead of the induced EMF. Only low-frequency currents can be used to accept output voltage instead of generated electromagnetic force because of their slight differences and the minimal influence of RC circuit characteristics. Errors in the output voltage’s magnitude and phase angle relative to the induced EMF can cause major errors in the reconstructed current when dealing with high-frequency currents. As a result, the reconstruction technique is developed using the coil’s measurable characteristics as a basis [88].

**Fig 8:**
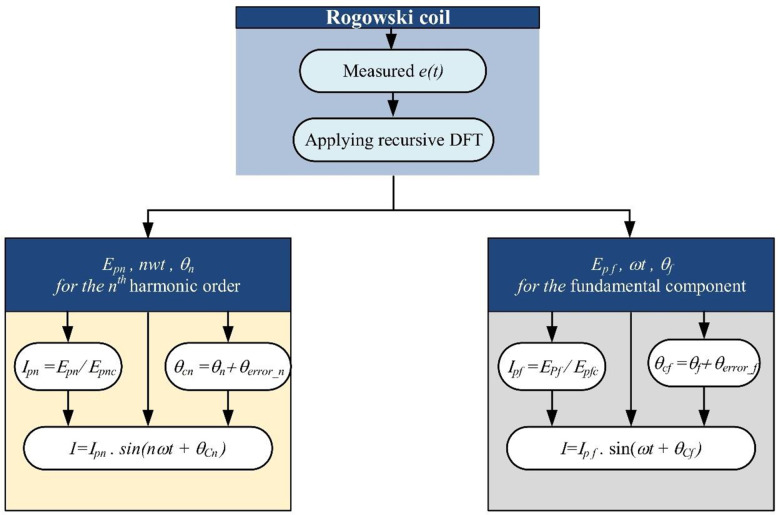
Computational engine process for current reconstruction.

By injecting a current of 1 A at various frequencies, from the fundamental value to the appropriate value for high-order harmonics, the measured characteristics of the coil can be determined. A calibrated current source is required for this technique to be carried out. The maximum output voltage value and the phase difference between the injected current and the output voltage waveforms (*ϕ*_*c*_, *θc*) are recorded at each frequency. Recursive DFT analysis is used to determine the maximum value and phase angle of the output voltage’s fundamental and harmonic components up to the required frequency. The output voltage is monitored at the RC terminals, as depicted in Fig 8. This is accomplished by adding the phase error of the measured characteristics to the phase angle at the corresponding frequencies and dividing the maximum value of each component by the maximum value recorded at the frequency corresponding to the measured coil characteristics. Once each frequency component’s maximum value and phase angle have been determined, a sine waveform can be constructed for each component. The total reconstructed current waveform can be obtained by summing these individual sine waveforms. This approach allows for the reconstruction of the input current waveform by analyzing the output voltage measured at the RC terminals, considering the measured characteristics of the coil, and using a recursive DFT to extract the individual frequency components and their corresponding phases, where *E*_*pn*_ denotes the peak value of induced the EMF at the order of the *n*th harmonics; *θ*_*n*_ denotes the phase angle at force at the order of the *n*th harmonics; *I*_*pn*_ denotes the peak value of CR at the order of the *n*th harmonics; *E*_*pn*_ denotes the peak value of induced electromotive force at the *n*th harmonics; *E*_*pnc*_ denotes the peak value of measured coil characteristic at the *n*th harmonics; *θ*_*cn*_ denotes the corrected phase angle at the order of the *n*th harmonics; *θ*_*error_n*_ denotes the phase angle error at the *n*th harmonics; *E*_*pf*_ denotes the peak value of induced electromotive force at the fundamental harmonic; *θ*_*f*_ denotes the phase angle at force at the fundamental harmonic; *I*_*pf*_ denotes the peak value of CR at the fundamental harmonic; *E*_*pf*_ denotes the peak value of induced electromotive force at the fundamental harmonic; *E*_*pfc*_ denotes the peak value of measured coil characteristic at the fundamental harmonic; *θ*_*cf*_ denotes the corrected phase angle at the fundamental harmonic; and the *θ*_*error_f*_ denotes the phase angle error at the fundamental harmonic.

### 4.2. Kalman computation engine

The power system voltage phases, current waveforms, and their harmonics are tracked over time using the Kalman filter [99]. The Kalman filter is based on a state space method, where the measurement (observation) equation of noisy observation signal models is modeled by the state equation, which also models the dynamics of the signal process. If a current signal *i*_*f*_(*t*) with amplitude *A*, frequency *ω*_0_, *θ* phase shift, DC decay of the amplitude denoted by *B*, and a time constant τ, then it can be written as follows:


$$i_{f}\left(t\right)\;=A\sin\left(\omega t+\theta \right)+Be^{-t/\tau }=x_{1}\cos\left(\omega t\right)+x_{2}sin\left(\omega t\right)+x_{3}e^{-t/\tau }$$ (15)

where *x*_*1*_=*Asin*(*θ*), *x*_*2*_ =*Acos*(*θ*), and *x*_*3*_ *=B*.

The input signal can be described as follows:


$$\begin{array}{c} x_{k+1}=\phi_{k}\cdot x_{k}+w_{k}\sqrt{b^{2}-4ac}\\ {\left[\begin{array}{l} x_{1}\\ x_{2}\\ x_{3} \end{array}\right]}_{k+1}=\left(\begin{array}{ccc} 1 & 0 & 0\\ 0 & 1 & 0\\ 0 & 0 & 1 \end{array}\right)\cdot {\left[\begin{array}{l} x_{1}\\ x_{2}\\ x_{3} \end{array}\right]}_{k}+{\left[\begin{array}{l} w_{1}\\ w_{2}\\ w_{3} \end{array}\right]}_{k} \end{array}$$ (16)

The equation of measurement includes both signal and noise as follows:


$$z_{k}=\left[\cos\left(\omega t_{k}\right)\sin\left(\omega t_{k}\right)e^{-\frac{t_{k}}{\tau }}\right]\left[\begin{array}{l} x_{1}\\ x_{2}\\ x_{3} \end{array}\right]+v_{k}$$ (17)

where *v*_*k*_ is the error due to noise or unmodeled harmonics.

This Kalman filter is only appropriate for the input signal specified in (15), presented in (16) and (17). The above state equations are solved using the steps in [99], to apply the Kalman filter approach. The samples available up to time *k* are used to predict the state. This is often stated as a linear combination of the innovation signal in *k* and the prediction based on samples available up to *k*-1. For their precise applications, like the initialization procedure vector, noise variation, and state variable covariance matrix, the characterization of state space equations and the choice of Kalman parameters are crucial challenges.

### 4.3. Least square computational engine

The combination of the collected trail curve of the measured samples taken at uniformly dispersed points at the appropriate time throughout a specific time window serves as the basis for the computational engine known as the least square (LS) for calculating the electrical phasors [100]. Compared to orthogonal algorithms, LS has the benefit of replicating periodic harmonic contents along with unknown fading parameters. The unknown time samples can be expressed as a function of time in the manner that follows given the *n*th harmonics:


e(t)=K1e−tτ+K1sinw0t+θ1+⋅⋅⋅+Knsinnw0t+θn (18)

Taylor series can be used and expressed in the exponential part e−tτ as follows:


$$e^{-\frac{t}{\tau }}=1-\frac{t}{\tau }+\frac{1}{2!}{\left(\frac{t}{\tau }\right)}^{2}-......................$$ (19)

The Taylor series terms are only taken into consideration for the first three terms, and therefore equation (18) is rewritten as follows:


$$e^{-\frac{t}{\tau }}=k_{1}-k_{1}\left(\frac{t}{\tau }\right)+\frac{k_{1}}{2!}{\left(\frac{t}{\tau }\right)}^{2}+k_{2}\sin\left(w_{0}t+\theta_{1}\right)+.....+k_{n}\sin\left(nw_{0}t+\theta_{n}\right)$$ (20)

Using a predetermined window length, with known sample *m* profiles, is determined for the signal as follows:


$$\left(\begin{array}{ccc} a_{11} & a_{12} & a_{1(2n+3)}\\ a_{21} & a_{22} & \vdots \\ a_{m1} & \cdots & a_{m(2n+3)} \end{array}\right)\left[\begin{array}{l} x_{1}\\ x_{2}\\ \vdots \\ x_{\left(2n+3\right)} \end{array}\right]=\left[\begin{array}{l} e(t_{1})\\ e(t_{2})\\ \vdots \\ e(t_{m}) \end{array}\right]$$ (21)

where *a*_11_, *a*_22_, …. are calculated based on the known constants of (16), while *x*_1_, *x*_2_, … are the unknown vector [*x*] to be estimated and are calculated for each sample as follows:


$$\left[x\right]=\left(\left[a^{t}\right]{\left[a\right]}^{-1}\right)\left[a\right]\left[e\right]$$ (22)

These estimated unknowns can be used to calculate the magnitudes and angles corresponding to the specified harmonics and the associated DC component.

### 4.4. Non-recursive DFT computational engine

The fundamental component of fault current signal samples *I*_*f*_ [*K*] can be extracted by applying a full-cycle DFT filter in its non-repeat form [101] according to the following formulas:


$$C\left[k\right]=\frac{2}{N}\overset{N-1}{\underset{n=0}{\sum }}i_{f}\left[n\right]\cos\left(\frac{2\pi n}{N}\right)$$ (23)

$$S\left[k\right]=\frac{2}{N}\overset{N-1}{\underset{n=0}{\sum }}i_{f}\left[n\right]\sin\left(\frac{2\pi n}{N}\right)$$ (24)

where *S* and *C* are the sine and cosine terms of the phasor and *n* is the sliding number over the sampling of the discrete windows of the current with the size of the window of samples *N*. *θ* is the sampling angle equal to *2π/N*. The magnitude of the peak value *I*_*f*_ [*K*] is given by:


$$I_{f}\left[k\right]=sqrt\left(C^{2}+S^{2}\right)$$ (25)

**Fig 9:**
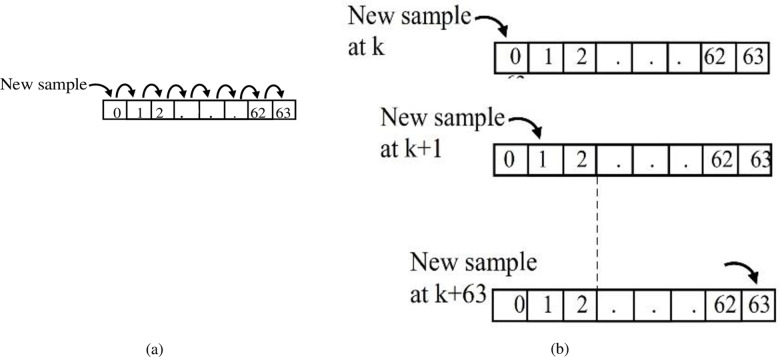
Sliding windows: **(a)** Commonly used sliding signal window, and (b) the sliding window for non-recursive DFT [56].

DFT is a relationship between a current sliding window and a base function window, where each window has *N* set to 200 samples, as depicted in Fig 9. Although the error in non-recursive DFT is fixed, its output angle is rotated, as the traditional window mechanism in Fig 9(a).

This behavior is achieved by rotating the window in advance before loading the new sample and considering that the same window uses the primary function at each step whose time reference is shifted by a time step when the signal window slides (or is updated by the new sample). Accordingly, this shift time produces an angle shift *Δθ =  2π/N*, as reported in [102], resulting in a non-stationary output phasor. A stationary non-recursive DFT is introduced to achieve non-rotated angle measurement, as shown in Fig 9(b). The signal window is updated by a new sample at each step, and the old sample is removed to keep the window size constant. A stationary phase angle can be attained by rotating the base function vector to achieve a base function with a fixed reference. Based on the method described above, further computations are required to rotate the base function or rotate the signal window. This is achieved by rotating the position of the new sample within the signal window. Specifically, the position of the new sample is not fixed at the first sample position as in traditional approaches. Instead, it gradually changes with each new sample, as shown in Fig 9(b). This rotating sample position idea saves computational time compared to the traditional approach of performing a full window rotation before loading new samples. In order to evaluate the engine, extensive tests are performed using computer simulation through the use of MATLAB simulation software, and the algorithm’s response to the fault current signal is recorded. However, for real-time evaluation, experimental tests of engines or relays are usually performed in the laboratory using the flexibility of digital computer simulations, as recently recommended by the IEEE committee. In this simulation, the current-wave forms and voltages taken from a computer simulation or digital fault recorder are converted into analog signals, amplified and then fed by tested hardware. This procedure would overcome the difficulties involved in fault tests that are impossible to perform on a real power system. The preparation of laboratory tests is described in the next section.

#### 4.4.1. Laboratory setup.

**Fig 10:**
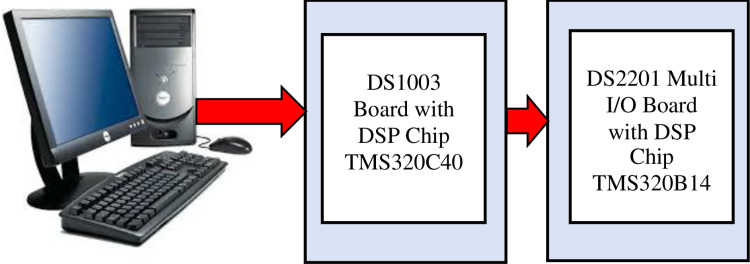
Block diagram of employed real-time engine devices.

The laboratory setup mainly consists of a DSP DS1003 board compatible with DS2201 multiplex board 110, as schematically shown in Fig 10. The DS1003 board is based on the floating-point DSP TMS320C40 Texas Instruments. The DS2201 facilitates interaction with the real system through 20 simultaneous analog input channels (distributed over 5 A/D switches), 8 analog output channels (distributed over 2 D/A switches), and 16-bit general-purpose 110-port ports. A personal computer (PC), was used as a host device for storing and downloading the software program. It also makes it easy to monitor and plot specific variables in real time using TRACE software. Non-recurring DFT is created depending on equations (23–25), considering the instructions for the DS 1003 software environment. In which. A high-level language develops the corresponding engine. Then. The software is compiled using Texas InstrumentsTMS320C40C-compiler/Assembler/Linker and then downloaded to DS 1003 Local and Global Memories. Below is a brief description of the main parts of the implemented algorithm

#### 4.4.2. Employed real-time engine.

The key parts of the executed real-time engine are the offline and online portions.

- Offline algorithm: where implemented devices must be initially assigned according to the configuration steps of the DSP board. Also, a specific sampling interval of 625 μ/s, which is equivalent to a sampling rate of 32 samples per cycle. This stage also includes the declaration of sine and cosine coefficients for DFT set to the core components of 50 Hz.
- Online calculation: This part of the algorithm is concerned with all the calculations performed simultaneously in each sample. It includes fault signal extraction, fault signal scanning, and non-recursive and variable tracking.
  - Signals in the program are defined either in (23-25) in a discrete or vector form for data. This data is usually extracted from power system simulation software such as Transient Electromagnetic Program (EMTP) or MATLAB. After that, it is minimized to fit the operating limits of the DS2201. The analog signals are then fed to the phase and executed on DS1003 devices.
  - A/D Conversion: The extracted error signal model MATLAB is converted through A/D converters to its digital form.
  - Estimator engine: Non-recurring DFT is performed according to (23-25). This engine is fed with the sampled fault signal.

One or all estimation engines can be enabled to evaluate the response in a comparative way

## 5. Simulation results for the classification process of the HIFs

The mathematical cores of the selected computational engine methods for detecting HIFs were constructed in MATLAB. Several fault scenarios were simulated in the simulation system to validate the effectiveness and reliability of the best-performing engine among the selected options under HIF conditions. The following illustrations showed the results when a fault occurred between buses 9–10 (at 865 m), as depicted in Fig 5. The prepared test investigated the performance of each engine, including the numerical arcing model and the numerical long arc model depicted in Fig 3. To visualize the performance of the selected engines, the profile of the estimated fundamental and different harmonic components of the fault current for each engine was characterized and compared with the recursive DFT approach already implemented in the protection relay mounted at the primary substation, as depicted in Fig 5. This comparative analysis allowed for the evaluation of the selected computational engines against the established recursive DFT method used in the existing protection system.

**Fig 11:**
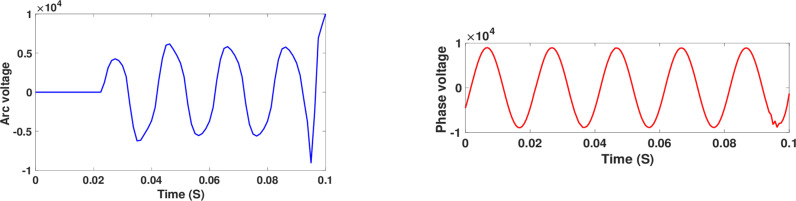
Voltage waveforms: (a) at the fault point, and (b) at the measuring point.

The fault point voltage is shown in Fig 11(a). The waveform is close to the square waveform. Accordingly, the fault point is a source of the third harmonic. Therefore, it is considered the feature extraction of the HIF case. Although it is not obvious in the voltage waveform at the measuring point, as shown in Fig 11(b). However, the harmonics due to the event of the fault are inherent in the measured waveforms, as evaluated in the following results.

**Fig 12:**
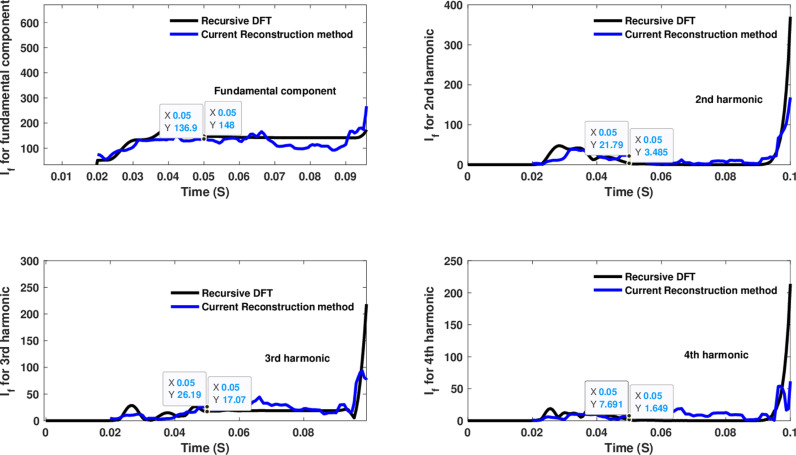
Estimated fundamental and 2nd, 3rd, and 4th harmonic orders of the fault current utilizing a CR-based engine.

In the first computational engine (current reconstruction), utilizing a numerical arc model, the different lumped parameters of the coils were implemented to obtain the best performance, as mentioned earlier. The parameters in this paper were selected, and the best results were given in [63], which indicates a high resonant frequency of 29 MHz. When a HIF is introduced using the numerical arc model at 0.02 seconds, Fig 12 shows large variations in the estimated fundamental, 2nd, 3rd, and 4th harmonic-order components of the utilized CR-based engine compared to the estimates of the same components using the recursive DFT approach. This variation is detected after one cycle when all components reach their peak values, which is used to detect the HIF. To provide a greater verification range for the efficiency of the selected computational engines, they were tested to calculate many harmonics up to the 20th order. This was done due to the exposure of distribution networks to nonlinear loads, such as *n*-pulse rectifier circuits or rectifiers, IGBT inverters, and capacitors in the distribution networks to improve the power factor. These capacitors carry out separation and connection operations during maintenance or faults. Additionally, the network contains loads such as induction motors that draw high currents, and the network is also exposed to the separation and connection operations of normal loads and changes in load levels (decrease or increase).

**Fig 13:**
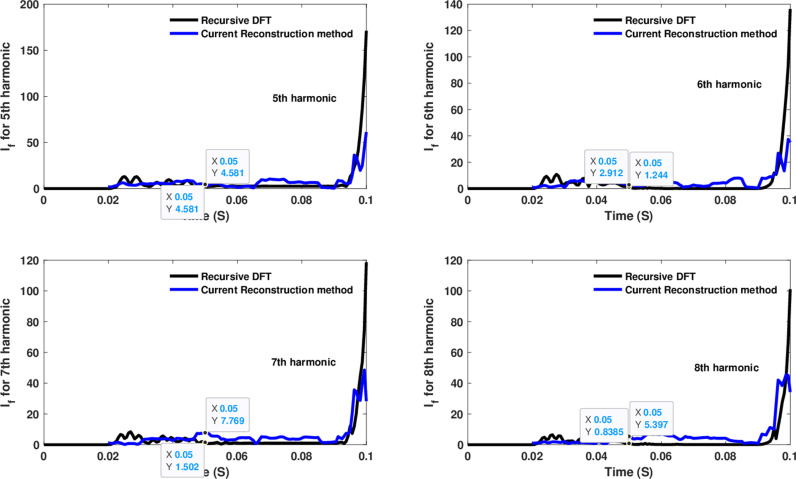
Estimated 5th, 6th, 7th, and 8th harmonic orders of the fault current utilizing a CR-based engine.

**Fig 14:**
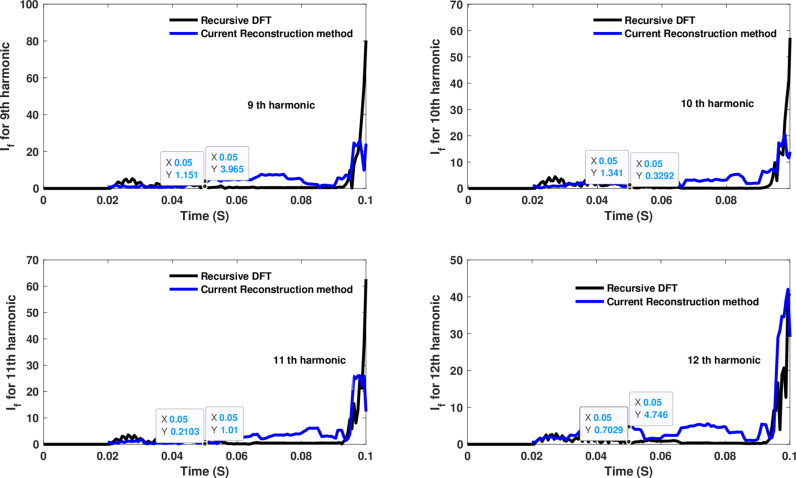
Estimated 9th, 10th, 11th, and 12th harmonic orders of the fault current utilizing CR-based engine.

**Fig 15:**
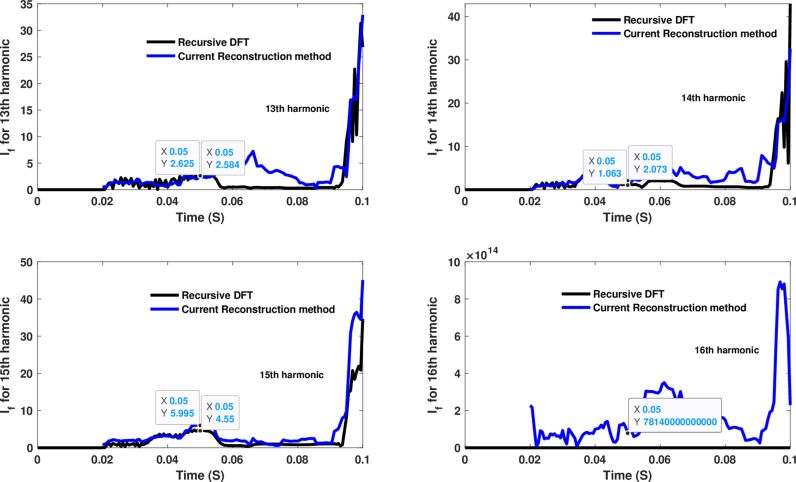
Estimated 13th, 14th, 15th, and 16th harmonic orders of the fault current utilizing CR-based engine.

**Fig 16:**
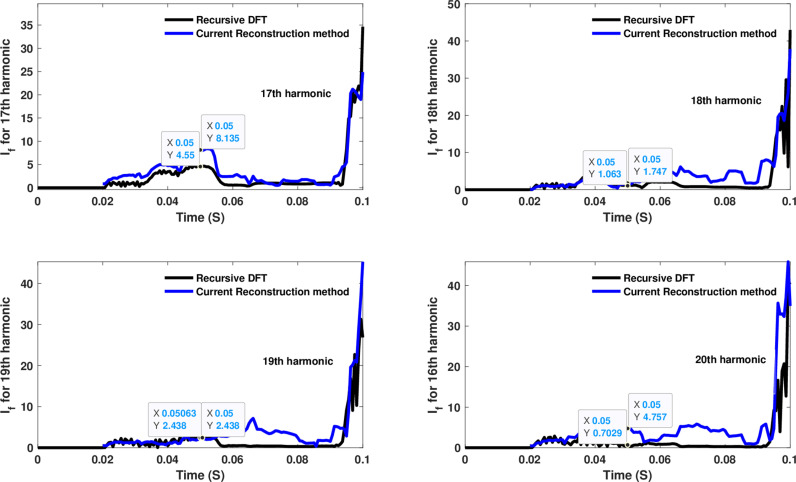
Estimated 17th, 18th, 19th, and 20th harmonic orders of the fault current utilizing CR-based engine.

Similarly, as shown in Figs 13–16 for the different harmonic orders investigated, the CR-based engine exhibited large variations in the estimated 5^th^ to 20^th^ harmonic order components of the fault current, compared to the recursive DFT approach. This comprehensive evaluation of harmonic components up to the 20th order further demonstrates the CR engine’s enhanced performance and detection capabilities under HIF conditions, where distribution networks are exposed to various nonlinear loads and operational scenarios.

**Fig 17:**
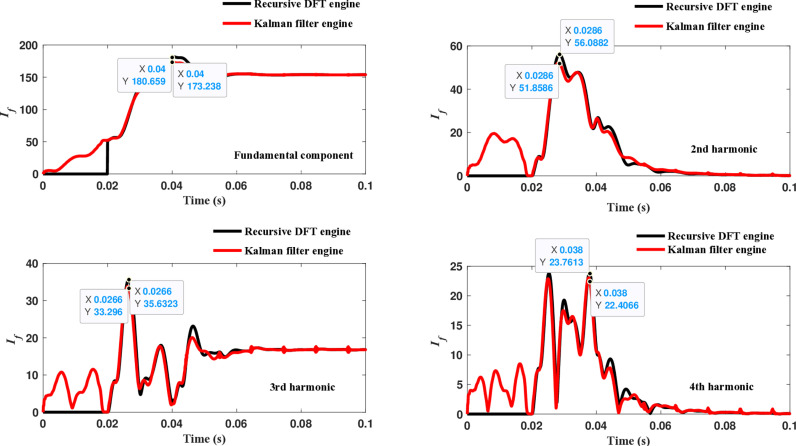
Estimated fundamental and 2nd, 3rd, and 4th harmonic orders of the fault current utilizing the Kalman filter engine.

**Fig 18:**
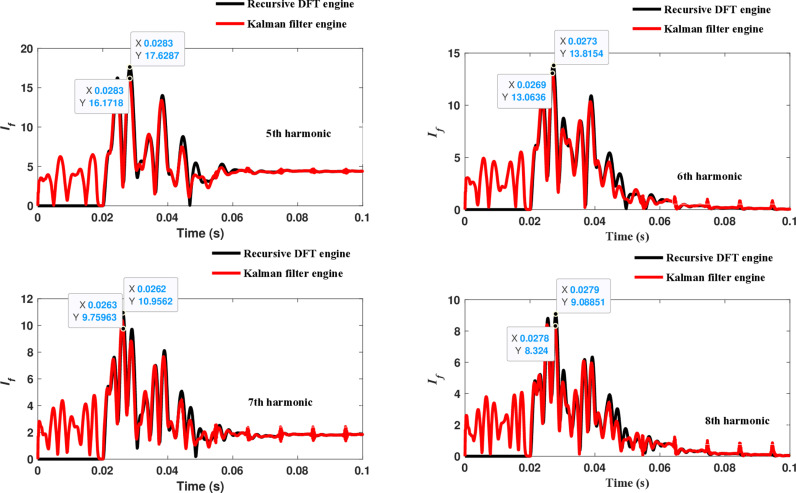
Estimated 5th, 6th, 7th, and 8th harmonic orders of the fault current utilizing the Kalman filter engine.

**Fig 19:**
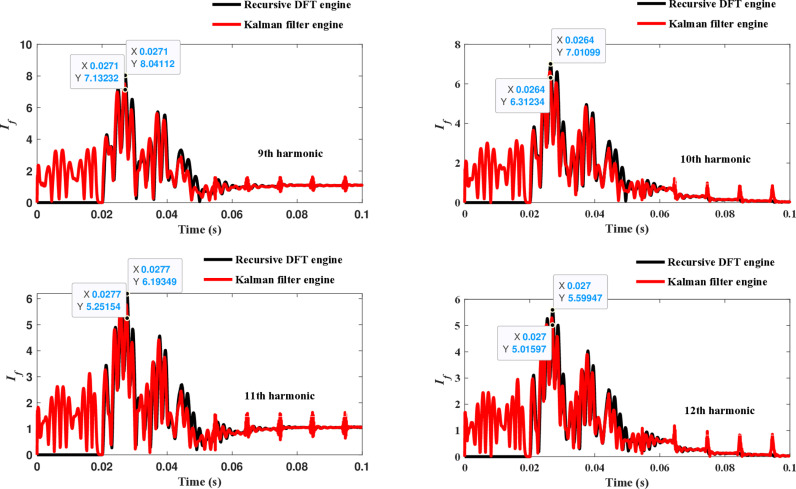
Estimated 9th, 10th, 11th, and 12th harmonic orders of the fault current utilizing the Kalman filter engine.

**Fig 20:**
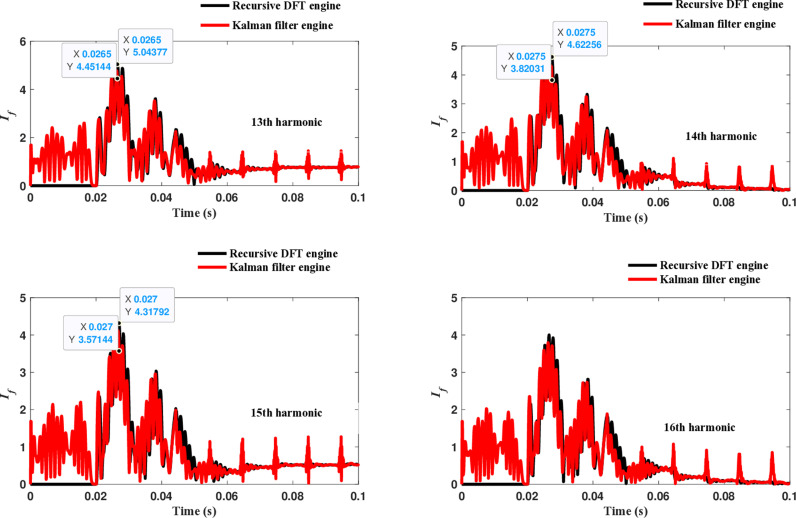
Estimated 13th, 14th, 15th, and 16th harmonic orders of the fault current utilizing the Kalman filter engine.

**Fig 21:**
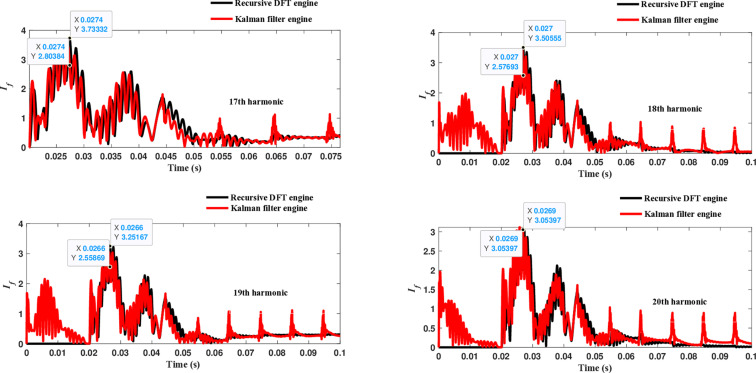
Estimated 17th, 18th, 19th, and 20th harmonic orders of the fault current utilizing the Kalman filter engine.

The second computational engine, the Kalman filter, utilizing the same numerical arc model, demonstrates the performance depicted in Fig 17. Compared to the recursive DFT approach, the Kalman filter performance exhibits relatively small variation in the estimated fundamental frequency as well as the 2^nd^ to 4^th^ harmonic-order components. However, as shown in Figs 18–21>> for the different harmonic orders investigated, the Kalman filter exhibits large variations in the estimated 5^th^ to 20^th^ harmonic-order components of the fault current compared to the recursive DFT. Additionally, the Kalman filter’s performance is not optimal initially, as it provides incorrect results during the first cycle and struggles to track the decreasing fault current rate accurately.

**Fig 22:**
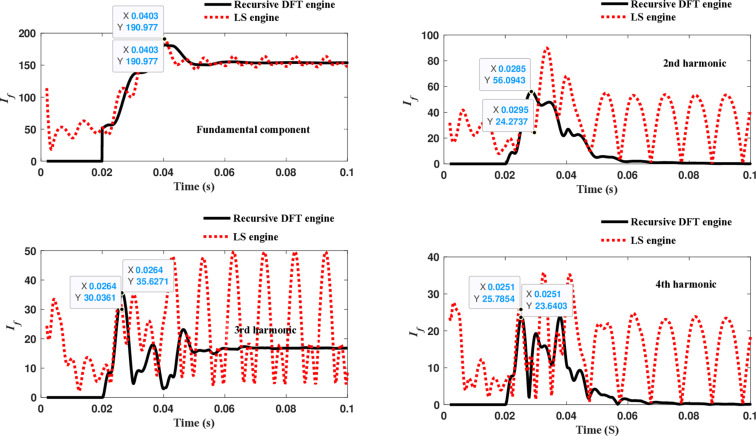
Estimated fundamental and 2nd, 3rd, and 4th harmonic orders of the fault current utilizing the LS-based engine.

**Fig 23:**
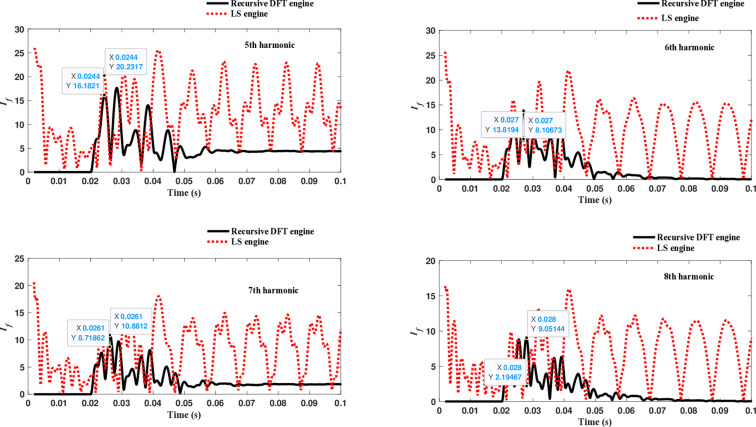
Estimated 5th, 6th, 7th, and 8th harmonic orders of the fault current utilizing the LS-based engine.

**Fig 24:**
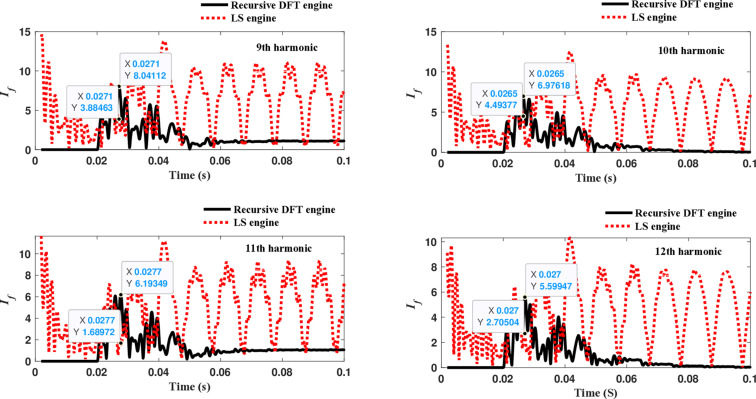
Estimated 9th, 10th, 11th, and 12th harmonic orders of the fault current utilizing the LS-based engine.

**Fig 25:**
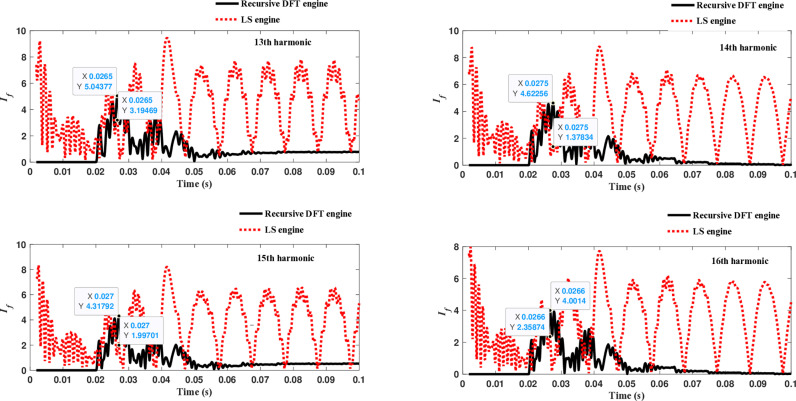
Estimated 13th, 14th, 15th, and 16th harmonic orders of the fault current utilizing the LS-based engine.

**Fig 26:**
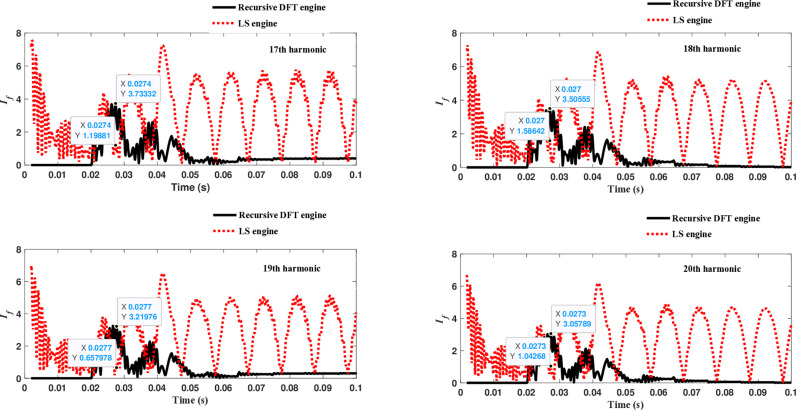
Estimated 17th, 18th, 19th, and 20th harmonic orders of the fault current utilizing the LS-based engine.

The third computational engine, the LS method, uses the same numerical arc model and offers good results in estimating the fundamental component. However, it does not perform equally well in calculating the 2^nd^ to 4^th^ harmonic-order components, as shown in Fig 22. Similarly, as illustrated in Figs 23–26 for the different harmonic orders investigated, the LS method exhibits large variations in the estimated 5^th^ to 20^th^ harmonic-order components of the fault current compared to the recursive DFT approach. This indicates that while the LS method performs well in estimating the fundamental component, it struggles to accurately capture the fault current’s higher-order harmonic content, which is crucial for robust HIF detection and classification.

**Fig 27:**
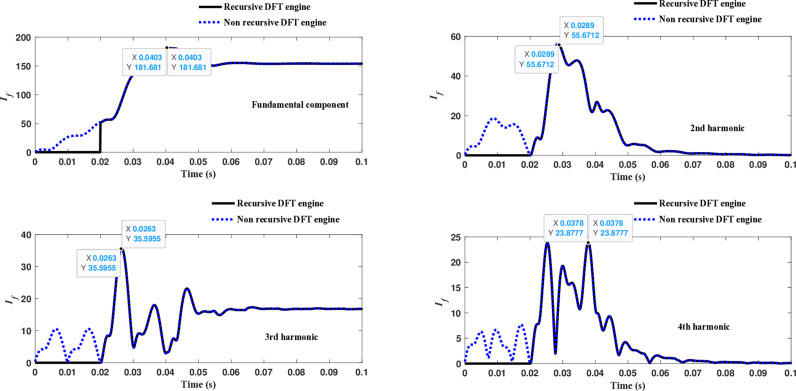
Estimated fundamental and 2nd, 3rd, and 4th harmonic orders of the fault current utilizing a non-recursive DFT-based engine.

**Fig 28:**
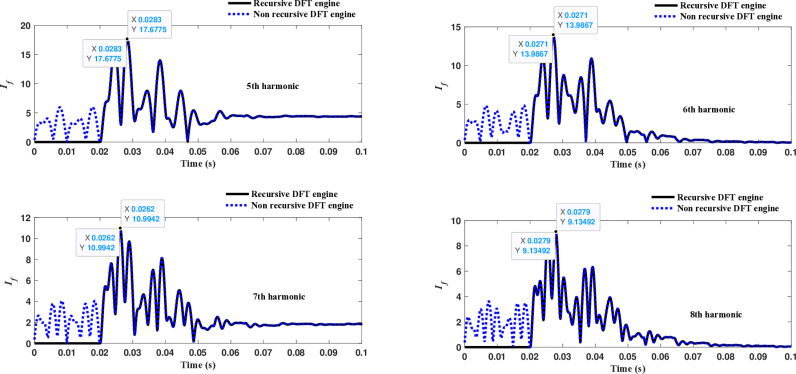
Estimated 5th, 6th, 7th, and 8th harmonic orders of the fault current utilizing a non-recursive DFT-based engine.

**Fig 29:**
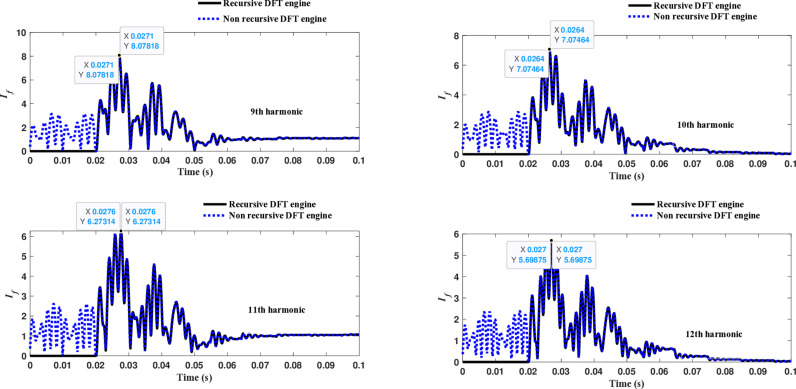
Estimated 9th, 10th, 11th, and 12th harmonic orders of the fault current utilizing a non-recursive DFT-based engine.

**Fig 30:**
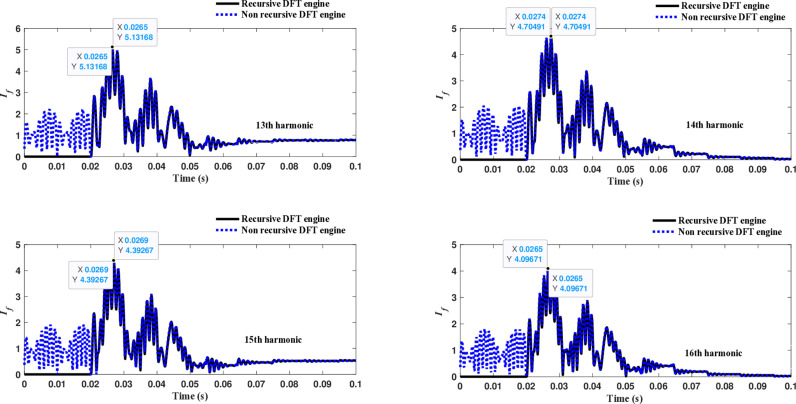
Estimated 13th, 14th, 15th, and 16th harmonic orders of the fault current utilizing a non-recursive DFT-based engine.

**Fig 31:**
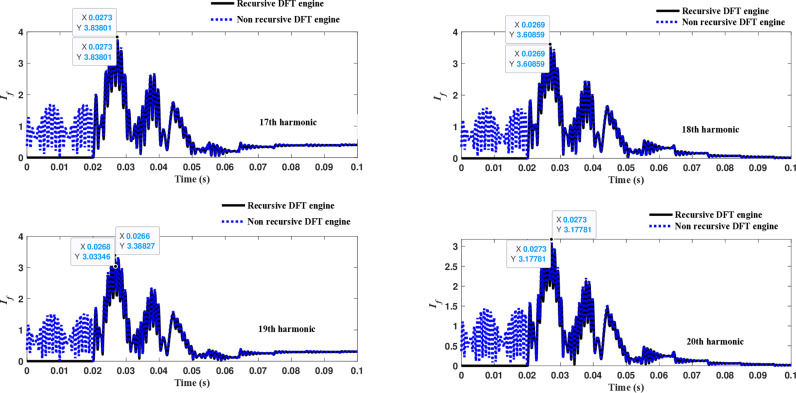
Estimated 17th, 18th, 19th, and 20th harmonic orders of the fault current utilizing a non-recursive DFT-based engine.

The fourth computational engine, the non-recursive DFT, uses the same numerical arc model and provides accurate results in estimating the fundamental component as well as the 2^nd^ to 4^th^ harmonic-order components, as shown in Fig 27. Similarly, as illustrated in Figs 28–31 for the different harmonic orders investigated, the non-recursive DFT approach exhibits precise results for the estimated 5^th^ to 20^th^ harmonic-order components of the fault current. This comprehensive evaluation demonstrates the superior performance of the non-recursive DFT computational engine in accurately estimating a wide range of harmonic components, which is crucial for effective HIF detection and characterization in distribution networks with diverse nonlinear loads and operating conditions.

## 6. Discussion

The analysis shows that using a non-recursive DFT reconstruction engine achieved accurate estimates of the fundamental and harmonic components up to the 20^th^ order. This engine demonstrated the best performance compared to the other computational approaches evaluated, such as the Kalman filter and LS methods. Based on these findings, the paper proposes the non-recursive DFT computational engine as an excellent approach for effective HIF detection and characterization in distribution networks. While the paper focused on testing the various computational engines using the numerical long arc model, the details of this model were not presented in the current paper. However, the authors note that all the computational engines were evaluated using this common numerical arc model as the basis for the analysis.

In this study, we evaluated the performance of several computational engines for detecting high-impedance faults (HIFs) in power distribution networks, with particular emphasis on numerical discrepancies in their effectiveness. Methods under comparison include non-recursive discrete Fourier transform (DFT), current reconstruction (CR) using Rogowski coils, Kalman filtration, and least squared computational motors

### 6.1. Non-Recursive DFT technique

The proposed non-recursive DFT method showed a clear advantage in terms of efficiency and computational accuracy. By using a fixed error in calculating capacity, it provides consistent results across different conditions. Numerical simulations revealed that this method maintains a lower error rate in estimating both basic and harmonic amplitudes compared to conventional iterative techniques. This stability is critical during transient conditions, where traditional methods often struggle with fluctuations.

### 6.2. Current reconstruction using rogowski coils

The current reconstruction method, although effective in many scenarios, showed insignificant numerical variation under different fault conditions. Simulation tests indicated that this approach is sensitive to the placement of Rogowski coils and ambient environmental factors, such as humidity and temperature. The model performance indicated a small percentage of errors in amplitude estimation, especially during active HIF conditions. This insensitivity can lead to consistency in fault detection, making it competitive and reliable compared with the proposed non-recurring DFT approach.

### 6.3. Kalman filtering

Kalman’s filtering provided a powerful framework for estimating system states, but the accuracy of initial conditions and model parameters heavily influenced its numerical performance. In scenarios with high noise levels, Kalman’s filter struggled to converge with real fault current values, resulting in greater estimation errors. Simulations have highlighted that while the filter can adapt over time, initial numerical estimates often have small skew results, particularly in transient fault conditions.

### 6.4. Least-squares computational engines

The least squares engine, although widely used for various signal processing applications, showed limitations in capturing the dynamic behavior of HIFs. Numerical assessments revealed that this technique tends to average the critical transient characteristics of fault signals, resulting in a higher overall error in the amplitude estimate. The comparative analysis indicated that although it is computationally less dense, it has less accuracy, especially in environments with rapidly changing fault conditions.

Numerical differences between methods emphasize the importance of choosing a suitable engine for detecting HIF in power distribution networks. The non-recurring DFT method not only outperformed other methods in terms of accuracy and stability but also provided a more reliable solution under various fault conditions. In summary, the non-recursive DFT reconstruction engine emerged as the superior approach for harmonic estimation and HIF detection, providing a promising solution for addressing the challenges associated with HIFs in modern distribution systems.

**Fig 32:**
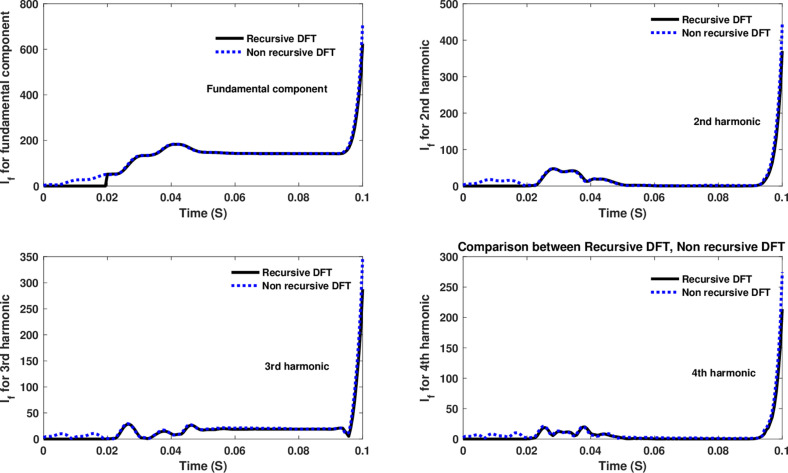
Estimated fundamental, 2^nd^, 3^rd^, and 4^th^ harmonic orders of the noisy fault current utilizing a non-recursive DFT-based engine.

**Fig 33:**
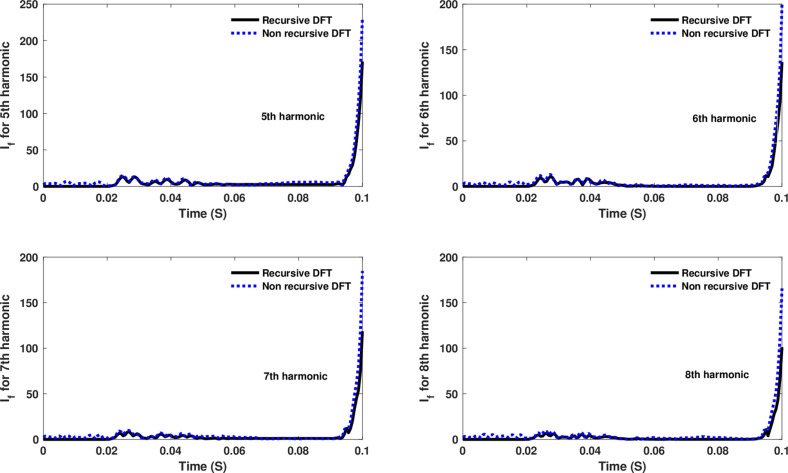
Estimated 5^th^, 6^th^, 7^th^ and 8^th^ harmonic orders of the noisy fault current utilizing a non-recursive DFT-based engine.

**Fig 34:**
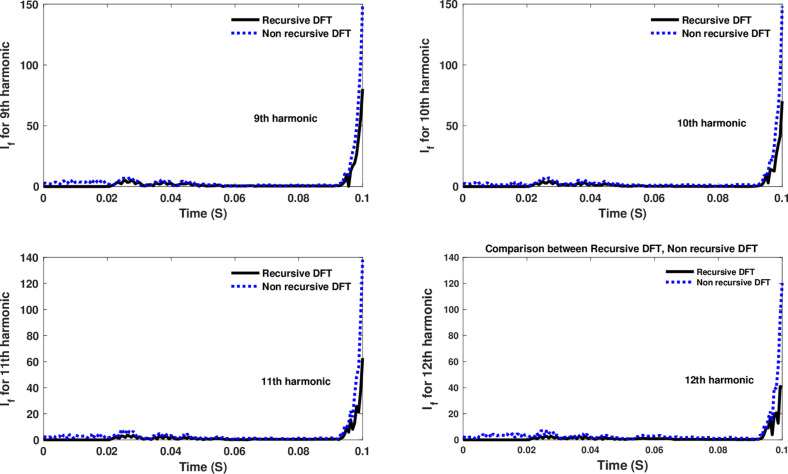
Estimated 9^th^,10^th^, 11^th^ and 12^th^ harmonic orders of the noisy fault current utilizing a non-recursive DFT-based engine.

**Fig 35:**
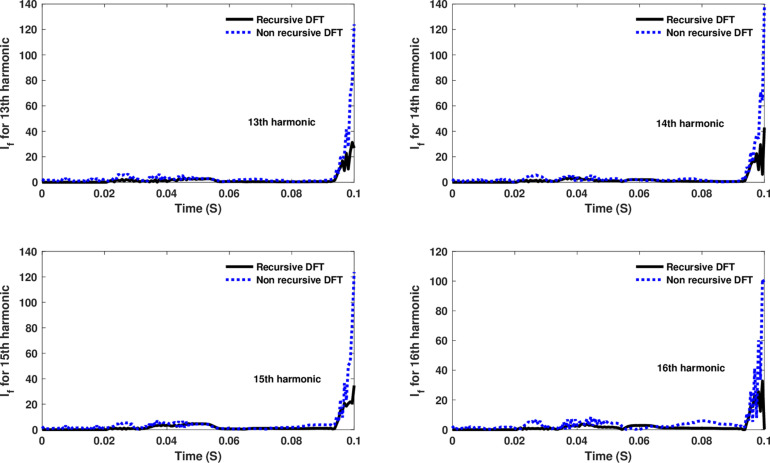
Estimated 13^th^,14^th^, 15^th^ and 16^th^ harmonic orders of the noisy fault current utilizing a non-recursive DFT-based engine.

**Fig 36:**
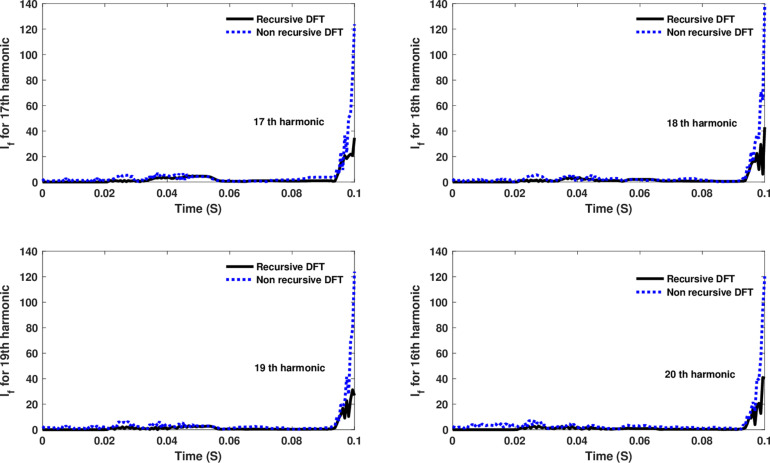
Estimated 17^th^,18^th^, 19^th^ and 20^th^ harmonic orders of the noisy fault current utilizing a non-recursive DFT-based engine.

Moreover, the noise impact on the suggested engine must be evaluated. Therefore, the recorded fault signals captured in the proposed engine are contaminated with white Gaussian noise, with a signal-to-noise ratio of 30–60 dB, as depicted in Figs 32–36 for the different harmonic orders investigated, utilizing the non-recursive DFT-based engine.

The results suggest that while noise introduces a certain level of distortion, its effect remains relatively small, particularly in the amplitude of signal calculated utilizing the proposed engine, as shown in Figs 32-36. The signal-to-noise ratio (SNR) refers to the preservation of the essential features of the signal despite the presence of noise, highlighting the durability of the proposed engine against minor disturbances. This flexibility is critical for applications that require precise signal processing, as it ensures that essential information remains

The proposed non-recursive DFT reconstruction engine is further validated by comparing its performance against the existing computational approaches, such as the current reconstruction, Kalman filter, and LS engines. This comparative assessment evaluates the percentage error between the reference current (obtained from the recursive DFT method) and the estimated current generated by each computational engine under consideration. The percentage error is calculated using equation (24), where *I*_*r*_ is the reference current’s peak, and *I*_*e*_ is the peak of the estimated current. Table 1 presents the fundamental and harmonics measurement errors for all computational engines investigated.

**Table 1:** Fundamental and harmonic measurement errors for all computational engines investigated.

Harmonic order	Error (%)			
**CR**	**Kalman filter**	**LS**	**Proposed**
**Fundamental**	1.034	4.5972	-5.2283	0.00482
**2** ^**nd**^	1.563	6.9492	-60.266	0.0268
**3** ^**rd**^	1.397	5.2124	-39.284	0.03004
**4** ^**th**^	1.524	3.3345	-49.895	0.10684
**5** ^**th**^	1.16	7.4023	-44.708	0.21171
**6** ^**th**^	1.112	6.2473	-57.237	0.37782
**7** ^**th**^	1.31	5.4065	-64.499	0.34639
**8** ^**th**^	1.341	7.4874	-75.27	0.51061
**9** ^**th**^	1.354	6.5392	-71.769	0.58415
**10** ^**th**^	1.118	5.6555	-78.175	0.50782
**11** ^**th**^	1.814	6.0365	-80.443	0.286
**12** ^**th**^	1.144	5.5689	-83.815	0.5396
**13** ^**th**^	1.943	5.4685	-87.175	0.5429
**14** ^**th**^	1.269	5.2169	-90.669	0.5816
**15** ^**th**^	1.048	4.6621	-89.998	0.5738
**16** ^**th**^	1.659	5.1907	-92.915	0.3821
**17** ^**th**^	1.224	4.321	-94.403	0.5044
**18** ^**th**^	1.949	2.1081	-95.971	0.5394
**19** ^**th**^	1.971	6.2555	-100.11	0.5011
**20** ^**th**^	1.378	-1.9224	-102.48	0.5215


$$e\left(\%\right)=\left(\frac{I_{r}-I_{e}}{I_{r}}\right)\cdot 100$$ (24)

As shown in Table 1, The CR engine exhibits a maximum error of 1.971% at the 19^th^ harmonic order. The Kalman filter engine performs better, with a maximum error of 7.4874% at the 8^th^ harmonic order. The LS engine demonstrates the worst performance, with a maximum error of -102.48% at the 20^th^ harmonic order.

In contrast, using the recursive and the proposed non-recursive DFT accurately tracks the reference currents measured. As presented in the table, the errors are significantly low, with a maximum value of only 0.58415% at the 9th harmonic order. This comprehensive comparative analysis conclusively demonstrates the proposed non-recursive DFT engine’s superior performance in estimating the fundamental and harmonic components of the fault current, outperforming existing computational approaches like current reconstruction, Kalman filter, and LS methods.

## 7. Comparative analysis with other published methods presented in the literature

The techniques discussed previously include the ML classifiers (ANN, artificial neural network; SVM, support vector machines; FLC, fuzzy logic controllers; ANFIS, adaptive neuro-fuzzy inference system; DTs, decision trees; GPR, Gaussian process regression; and others), the provided measurement signals, and feature extraction methods. All possess distinctive capabilities for the detection (DT), classification (CS), and localization (LT) of HIFs. Building upon this foundation, a comparative analysis is presented in Table 2. The comparison is based on using accuracy as a metric for the performance evaluation of these techniques. Additionally, security (*S*) and dependability (*D*) considerations are investigated, which can be quantified through the accuracy ratio of HIF fault diagnosis, an aspect often overlooked in the existing literature. Furthermore, the selected and proposed computational engines are also analyzed using these criteria. Table 2 indicates that the proposed non-destructive DFT approach could offer advantages in terms of data acquisition and processing compared to other techniques. The proposed method achieves an accuracy of 99.61%, among the highest values reported in the table. This high accuracy level indicates the effectiveness of the proposed technique in accurately detecting and classifying HIFs. The proposed method’s security and dependability are reported as 99.61%. These high values suggest that the proposed technique provides a robust and reliable solution for HIF detection, with a low risk of misdiagnosis or security vulnerabilities. This means that the use of a non-destructive DFT approach in the proposed method may offer several advantages, such as reduced impact on the power system during data acquisition, as non-destructive techniques typically have minimal interference, improved computational efficiency and real-time processing capabilities, as DFT-based analyses can be optimized for faster execution, and potential for integration with existing power system monitoring and control infrastructure, leveraging the widespread use of DFT-based techniques in the industry.

**Table 2:** Accuracy, dependability, and security assessment of several methods.

Ref.	Measurement	Technique used	ML Classiﬁers	Objectives	Accuracy (%)	*D* (%)	*S* (%)
[31]	Voltage (V) & current (I)	WT	SVM	DT	91.38	90.04	92.6
[103]	I	DFT	ANFIS	DT, CS	99.64	–	–
[32]	Arc voltage	–	ANN	DT	99.35	–	–
[35]	Resistance	EMD	ANN	LT	99.00	–	–
[33]	V & I	WT	ANN	DT	91.33	–	–
[104]	V & I	WT	ANN	DT	95.989	–	–
[105]	I	WT	SVM	DT, CS	96.00	–	–
[106]	I	Variational mode decomposition	SVM	DT, CS	99.00	–	–
[107]	I	Teager energy operator	Fuzzy inference system	DT, CS	–	–	–
[51]	V & I	WT	FLC	CS	88.89	–	–
[108]	I	Mathematical morphology	DTs	DT	99.34	100.00	98.77
[57]	I	FFT	FLC	DT	–	–	–
[49]	I	WT	ANN	CS	–	–	–
[109]	V & I	ST	ANN	DT	95.43	–	–
[110]	I	Morphology gradient	FLC	DT	99.40	99.78	99.07
[111]	V & I	–	SVM	DT	–	100.00	100.00
[112]	I	WT	FLC	LT	99.24	–	–
[53]	V & I	–	ANN	LT	99.67	–	–
[113]	I	Mathematical morphology	DTs	DT	98.33	98.88	100.00
[114]	V & I	WT	SVM	LT	99.34	–	–
[115]	V & I	WT	ANN	DT	–	–	–
[116]	I	WT	ANN and GPR	LT	99.40	–	–
[117]	I	–	FLC	DT, CS	–	–	–
[118]	I	WT	ANN	DT, CS	99.00	–	–
[54]	V & I	WT	ANN	DT	96.00	–	–
[119]	I	WT	SVM	DT	99.00	–	–
[120]	I	WT	DTs	DT	98.22	95.79	100.00
[121]	V & I	–	ANFIS	LT	99.25	–	–
[122]	I	WT	Extreme learning machine	DT	–	–	–
[123]	I	WT	Self-organizing mapping network	LT	91.27	–	–
[124]	I	ST	ANN	LT	99.15	–	–
[55]	I	ST	Extreme learning machine	DT, CS	99.3	–	–
**CR**	I	CR	–	DT	88.38	90.00	90.00
**Kalman filter**	I	Kalman filter	–	DT	86.94	90.00	90.00
**LE**	I	LE	–	DT	Not acceptable	5	5
**Proposed**	I	Non-recursive DFT	–	DT	99.61	99.61	99.61

## 8. Conclusion

### 8.1. Summary of the paper

A novel computational engine using a non-recursive DFT approach was presented to measure AC currents’ fundamental and high-order harmonic components accurately. This capability is crucial for effectively modeling and detecting HIFs in power distribution networks. The paper first discussed and evaluated different HIF modeling techniques, selecting the most suitable approaches for further analysis. A selected technique, the CR engine, was implemented in MATLAB/Simulink for comparative assessment. A specialized coil sensor was also developed to assess power-frequency distorted currents with low-order and high-order harmonics. Three computational engines, the CR, Kalman Filter, and LS methods, were then utilized for comparative evaluation. All the computational engines, including the proposed non-recursive DFT approach, were implemented and evaluated using MATLAB/Simulink. The simulation study focused on measuring HIF conditions in the first section of the selected power system, which represents the most challenging scenario due to the varying power ratings at the feeder origin. The comparative analysis conclusively demonstrated the proposed Non-recursive DFT engine’s superior performance. It exhibited the lowest percentage error between the reference current (from the recursive DFT) and the estimated current, outperforming the existing computational approaches like CR, Kalman Filter, and LS methods.

Furthermore, the proposed non-recursive DFT engine’s efficacy was validated against recently published HIF detection and characterization techniques, further corroborating its effectiveness in accurately estimating fault currents’ fundamental and harmonic components. Finally, the presented Non-recursive DFT computational engine provides a robust and reliable solution for HIF modelling, detection, and characterization in power distribution networks, with significant performance improvements over several state-of-the-art methods. The suggested computation engine has a wide range of consequences for fault detection. In addition to improving safety and compliance, they also result in significant cost savings and higher operational effectiveness.

### 8.2. The main contribution

This paper makes several important contributions to the field of high-impedance fault detection in power distribution networks:

- A new computational engine: We introduce a non-recursive computational engine for discrete Fourier transform (DFT), which improves the accuracy of fault detection compared to traditional methods.
- Improved detection capabilities: Our technology demonstrates a significant reduction in the estimated error percentage for detecting fundamental and harmonic amplitudes, outperforming conventional methods by up to 0.39%. Integration with existing systems: The proposed engine can be easily integrated into existing protection migration systems and does not require any additional hardware, thus reducing implementation costs.
- Real-world validation: Comprehensive simulations with the IEEE 33 carrier test -bus verify the effectiveness of our engine under various fault conditions, providing confidence in its real-world applicability.
- Framework for future research: We identify key areas for further investigation, such as exploring adaptive algorithms that can enhance detection accuracy in dynamic load conditions.
- By addressing these aspects, we aim to contribute to the development of more reliable and efficient fault detection systems in power distribution networks.

### 8.3. Deficiencies and prospects

- Applicability: The suggested computational engine may be very applicable in practical situations when different environmental factors influence the nature of faults. For example, varying soil moisture content and resistivity can affect fault currents, which may result in inaccurate detection.
- Noise issues: One important concern is the sensitivity of the suggested computational engine to noise. Faulty detection of faults may arise from the deterioration of the signal quality current caused by external electromagnetic interference and fluctuations in the power system. In high noise conditions, conventional noise reduction technologies may not be sufficient, which can compromise detection reliability.
- Performance: While the proposed method outperforms conventional techniques in many simulations, its performance under diverse operational conditions, such as load variations and system configurations, remains untested. Differences in transient behaviors can lead to complications that the current model may not adequately address.

### 8.4. Future directions

- Improved modeling: Future research should focus on developing more sophisticated models that incorporate environmental variables, such as soil conditions and moisture, to improve the technology’s adaptability.
- Integrating machine learning algorithms can allow the system to learn from varied datasets and adapt to different conditions.
- Noise mitigation strategies: Implementing advanced signal processing techniques, such as adaptive filtering or wavelet conversions, can help mitigate noise effects. Research into the integration of these methods with existing non-iterative DFT can enhance signal clarity and accuracy.
- Field testing: Conducting extensive field tests in various real-field conditions can provide valuable insights into the performance of technology. Evaluating the effectiveness of the computational engine in different distribution networks and fault scenarios can help determine its limits and areas for improvement
- Hybrid methods: Exploring hybrid methods that combine non-recursive DFT with other detection methods, such as AI-based algorithms, may lead to better results. These methods can leverage the strengths of multiple engines to enhance the reliability of fault detection.
- User training and system integration: The development of guidelines for training users in the operational context of the system can improve effectiveness. Moreover, integrating this engine with existing protective migration systems can simplify their implementation and ensure better fault management.
- Real-time data from various environments and fault types could provide insights into the model’s robustness and adaptability, and field tests would allow the assessment of the model’s performance in practical applications, ensuring reliability and accuracy in detecting high-impedance faults in power distribution networks. To further refine the model and improve fault detection methodologies, future research could concentrate on improving the proposed model by integrating additional data sources and conducting field tests.

Addressing these limitations through targeted research and development will enhance the durability and applicability of non-recurring DFT technology for high-impedance fault detection, ultimately contributing to safer and more reliable power distribution networks.

## Supporting Information

S1 File Selected feeder parameters. Line impedance and load power”.
(DOCX)
