# Supplementary material for: High impedance faults detection in power distribution networks using rogowski coils, kalman filtering, least-squares and non-recursive DFT computation engines
Source: PLoS One. 2025 Apr 17;20(4):e0320125. doi: 10.1371/journal.pone.0320125 (PMC12005528; doi:10.1371/journal.pone.0320125)
Supplement: S1 File — Line impedance and load power”. (DOCX) [file pone.0320125.s001.docx]

S1 File. Selected feeder parameters. Line impedance and load power

Table A.1 presents the selected feeder parameter. The zero-sequence resistance per positive-sequence resistance of the feeder is 1.62, while the zero-sequence inductance per the positive-sequence inductance is 3.82. The positive and zero sequence capacitances are 0.0067 and 0.0041 μF/km, respectively.

Table A.1. Selected feeder parameters.

| Bus | Line impedance | | Load power | |
| --- | --- | --- | --- | --- |
|  | *R*_1_ (Ω) | *X*_1_(Ω) | *P_L_* (kW) | *Q_L_* (kVAR) |
| 2 | 0.0922 | 0.0470 | 100.0 | 60.0 |
| 3 | 0.4930 | 0.2511 | 90.0 | 40.0 |
| 4 | 0.3660 | 0.1864 | 120.0 | 80.0 |
| 5 | 0.3811 | 0.1941 | 60.0 | 30.0 |
| 6 | 0.8190 | 0.7070 | 60.0 | 20.0 |
| 7 | 0.1872 | 0.6188 | 200.0 | 100.0 |
| 8 | 0.7114 | 0.2351 | 200.0 | 100.0 |
| 9 | 1.0300 | 0.7400 | 60.0 | 20.0 |
| 10 | 1.0040 | 0.7400 | 60.0 | 20.0 |
| 11 | 0.1996 | 0.0650 | 45.0 | 30.0 |
| 12 | 0.3744 | 0.1238 | 60.0 | 35.0 |
| 13 | 1.4680 | 1.1550 | 60.0 | 35.0 |
| 14 | 0.5416 | 0.7129 | 120.0 | 80.0 |
| 15 | 0.5910 | 0.5260 | 60.0 | 10.0 |
| 16 | 0.7463 | 0.5450 | 60.0 | 20.0 |
| 17 | 1.2890 | 1.7210 | 60.0 | 20.0 |
| 18 | 0.7320 | 0.5740 | 90.0 | 40.0 |
| 19 | 0.1640 | 0.1565 | 90.0 | 40.0 |
| 20 | 1.5042 | 1.3554 | 90.0 | 40.0 |
| 21 | 0.4095 | 0.4784 | 90.0 | 40.0 |
| 22 | 0.7089 | 0.9373 | 90.0 | 40.0 |
| 23 | 0.4512 | 0.3083 | 90.0 | 50.0 |
| 24 | 0.8980 | 0.7091 | 420.0 | 200.0 |
| 25 | 0.8960 | 0.7011 | 420.0 | 200.0 |
| 26 | 0.2030 | 0.1034 | 60.0 | 25.0 |
| 27 | 0.2842 | 0.1447 | 60.0 | 25.0 |
| 28 | 1.0590 | 0.9337 | 60.0 | 20.0 |
| 29 | 0.8042 | 0.7006 | 120.0 | 70.0 |
| 30 | 0.5075 | 0.2585 | 200.0 | 600.0 |
| 31 | 0.9744 | 0.9630 | 150.0 | 70.0 |
| 32 | 0.3105 | 0.3619 | 210.0 | 100.0 |
| 33 | 0.3410 | 0.5302 | 60.0 | 40.0 |
